# Biocontrol Activity of Nonpathogenic Strains of *Fusarium oxysporum*: Colonization on the Root Surface to Overcome Nutritional Competition

**DOI:** 10.3389/fmicb.2022.826677

**Published:** 2022-01-27

**Authors:** Yuichiro Iida, Aya Ogata, Hiroki Kanda, Oumi Nishi, Hirotoshi Sushida, Yumiko Higashi, Takashi Tsuge

**Affiliations:** ^1^National Agriculture and Food Research Organization, Tsu, Japan; ^2^Graduate School of Bioagricultural Sciences, Nagoya University, Nagoya, Japan; ^3^Laboratory of Plant Protection and Biotechnology, Kinki University, Nara, Japan

**Keywords:** Fusarium wilt disease, biocontrol, nonpathogenic *Fusarium oxysporum*, *F. oxysporum* f. sp. *melonis*, *F. oxysporum* f. sp. *lycopersici*, pathogenicity mutant, nutrient competition, rhizosphere

## Abstract

*Fusarium oxysporum* is a soil-borne fungal pathogen that causes vascular wilts in a wide variety of crops. Certain nonpathogenic strains of *F. oxysporum* are known to protect crops against *F. oxysporum* pathogens. We assessed the biocontrol activities of nonpathogenic mutants of *F. oxysporum* ff. spp. *melonis* and *lycopersici* generated by disruption of the *FOW2* gene, which encodes a Zn(II)2Cys6-type transcriptional regulator essential for their pathogenicity. Pre-inoculation of melon or tomato roots with strain Δ*FOW2* conidia markedly reduced disease incidence caused by the parental wild-type strain in a concentration-dependent manner of conidial suspensions of Δ*FOW2* strains. The biocontrol effect caused by the Δ*FOW2* pre-inoculation lasted for at least 7 days. Pre-inoculation of melon roots with the wild-type or Δ*FOW2* strain of *F. oxysporum* f. sp. *lycopersici* and nonpathogenic *F. oxysporum* strain also led to biocontrol activity against *F. oxysporum* f. sp. *melonis*, indicating that the biocontrol activity of Δ*FOW2* strains is due to its nonpathogenic nature, not to the *FOW2* disfunction. Conidial germination and hyphal elongation of only the wild-type strain were inhibited on melon root surface pre-inoculated with conidia of strains nonpathogenic to melon plants. Expression of defense-related genes was not significantly induced in roots and aboveground parts of melon seedlings preinoculated with Δ*FOW2* conidia. Carbon source competition assay showed that nonpathogenic strains competed with the wild-type strain for a carbon source in soil. Strain Δ*FOW2* also competed with the oomycete pathogen *Pythium aphanidermatum* for carbon source and protected melon plants from *P. aphanidermatum.* Our results suggest that the biocontrol activity of the nonpathogenic *F. oxysporum* strains used in this study mainly depends on their extensive colonization of the root surface and outcompeting pathogens for nutrients.

## Introduction

The soil-borne pathogen *Fusarium oxysporum* is a facultative fungus that causes economically important losses in a wide range of crops (Michielse and Rep, 2009; Edel-Hermann and Lecomte, 2019). Intraspecific variants of the fungus, called formae speciales (f. sp.), cause wilting symptoms (Fusarium wilt disease) on over 100 plant species. Hyphae of *F. oxysporum* penetrate the roots and invade the vascular system during the infection. Although the most practical control methods for this disease involve soil fumigation with chemicals and the use of resistant cultivars, the chemicals (e.g., trichloronitromethane and methyl bromide) have adverse effects on human and the environment, and new fungal races often emerge that can overcome resistant cultivars (Michielse and Rep, 2009). Some isolates cause Fusarium wilt disease on crops, but most strains are nonpathogenic soil saprophytes, and pretreatment of plants with some of these nonpathogenic strains often suppresses Fusarium wilt disease (Rouxel et al., 1979; Fravel et al., 2003; Alabouvette et al., 2009).

The idea of using nonpathogenic *F. oxysporum* to control Fusarium diseases came from studies of soils naturally suppressive to Fusarium wilts (Toussoun, 1975; Louvet et al., 1976). High populations of nonpathogenic *F. oxysporum* and *F. solani* in suppressive soils contribute to the suppressive effect (Rouxel et al., 1979), but nonpathogenic strains of *F. oxysporum* are much more effective than strains of other *Fusarium* species (Tamietti and Alabouvette, 1986). Interactions between pathogenic and nonpathogenic *F. oxysporum* strains in suppressive soils directly or indirectly contribute to disease control; therefore, nonpathogenic strains have been developed as biocontrol agents (Ogawa and Komada, 1984; Postma and Rattink, 1992; Alabouvette et al., 1993). Their main modes of action include competition for nutrients and trace elements in the rhizosphere and for infection sites on the root surface and induction of plant resistance (Ogawa and Komada, 1984; Postma and Luttikholt, 1996; Fuchs et al., 1997; Larkin and Fravel, 1998; Fravel et al., 2003; Alabouvette et al., 2009). These mechanisms vary in importance depending on the strain.

The endophytic strain Fo47, originally isolated from the suppressive soil, is one of the best-studied biocontrol agents (Alabouvette, 1986). Fo47 colonizes the root surface and the soil near root epidermal cells and competes for nutrients with pathogens (Larkin and Fravel, 1999; Olivain et al., 2003; Bolwerk et al., 2005). In addition, Fo47 induces plant defense responses but not via well-known defense pathways such as salicylic acid, jasmonic acid, ethylene, and pattern-triggered immunity (Constantin et al., 2019; de Lamo et al., 2020). Another well-studied nonpathogenic biocontrol strain of *F. oxysporum*, CS-20, triggers defense responses more strongly than Fo47, which correlates with CS-20 being the more potent biocontrol agent (Lemanceau et al., 1993; Larkin and Fravel, 1999; Bolwerk et al., 2005). Biocontrol strain MSA35 grows in association with a consortium of exogenous bacteria that inhibit mycelial growth and expression of pathogenicity genes in pathogenic *F. oxysporum*, and when “cured” of the bacteria, was identified as *F. oxysporum* f. sp. *lactucae* (Minerdi et al., 2008). MSA35 secretes volatile organic compounds into the soil, which reduce mycelial growth of pathogenic *F. oxysporum* strains (Minerdi et al., 2009). One of these volatiles, the sesquiterpene α-humulenone, represses the expression of pathogenicity genes in *F. oxysporum*. Thus, the biocontrol activity of nonpathogenic *F. oxysporum* strains is due to different mechanisms and sometimes to a combination of mechanisms. However, the molecular basis of the biocontrol potency of particularly effective strains is still far from being understood.

Many genes involved in pathogenicity of *F. oxysporum* have been identified (see review by Husaini et al., 2018). A mitogen-activated protein kinase (*FMK1*) (Di Pietro et al., 2001) and G protein subunits α (*FGA1*) and β (*FGB1*) (Jain et al., 2002, 2003) are required for normal germination of conidia of *F. oxysporum*. An F-box protein (*FRP1*) (Duyvesteijn et al., 2005) is indispensable for colonization on the root surface, and a putative β-1,3-glucanosyltransferase gene (*GAS1*) (Caracuel et al., 2005) appears to be essential for colony growth on solid substrates and for invasive growth in host tissue. A fungal-specific Zn(II)2Cys6-type transcriptional regulator Fow2 (Imazaki et al., 2007), is required for invasion of the root tissue and host colonization, but is dispensable for vegetative growth, conidiation, and the use of carbon sources. *FOW2* is widely conserved in formae speciales of *F. oxysporum* (Imazaki et al., 2007), whereas the genes regulated by Fow2 have not been identified. Knockout mutants of these pathogenicity genes have been reported to be nonpathogenic or have markedly reduced virulence (Di Pietro et al., 2001; Jain et al., 2002, 2003; Caracuel et al., 2005; Duyvesteijn et al., 2005; Imazaki et al., 2007). In this study, we investigated the biocontrol activity of these six mutants that have various defined characteristics at different stages of infection on the host roots. We compared the biocontrol activity of pathogenicity mutants, nonpathogenic *F. oxysporum*, and avirulent *F. oxysporum* f. sp. *lycopersici* as nonpathogenic strains against *F. oxysporum* f. sp. *melonis* after inoculation of melon plants. Hyphal growth of *F. oxysporum* strains on the root surface was observed with a confocal laser scanning microscope, and the ability of the strains to induce resistance was evaluated. The biocontrol effect of pathogenicity mutants of *F. oxysporum* was also verified against the soil-borne oomycete *Pythium aphanidermatum*, which causes damping-off and root rot diseases of cucurbitaceous plants.

## Materials and Methods

### Fungal and Oomycete Strains

Fungal and oomycete strains used in this study are listed in Table 1. *F. oxysporum* f. sp. *melonis* strain Mel020120 and *F. oxysporum* f. sp. *lycopersici* strain CK3-1 were used as the pathogenic wild types (Namiki et al., 1994). Nonpathogenic *F. oxysporum* strain MFG6, a biocontrol agent against the strawberry pathogen *F. oxysporum* f. sp. *fragariae*, was kindly provided by Dr. Katsutoshi Kuroda (Mie Prefecture Agricultural Research Institute, Japan) (Kuroda et al., 2004). Knockout mutants and green fluorescent protein (GFP)-transformed strains were generated from these strains. For preparing conidia, strains were cultured in potato dextrose broth (PDB) (BD, Detroit, MI, United States) at 25°C for 3 to 4 days with shaking in the dark. The resulting conidia were collected by centrifugation, washed and resuspended in sterilized water.

**TABLE 1:** Fungal strains used in this study.

Strain	Description	References
Mel02010	*F. oxysporum* f. sp. *melonis*	Inoue et al., 2001
Mel02010-DsRed	DsRed-expressing Mel02010	
Mel02010-DsRed^TR^	Thiophanate-methyl resistant Mel02010-DsRed	
MF2-1 and MF2-2	Δ*FOW2* Mel02010	Imazaki et al., 2007
MF2-1-GFP	GFP-expressing MF2-1	Imazaki et al., 2007
GA1 and GA2	Δ*FGA1* Mel02010	
GB1 and GB2	Δ*FGB1* Mel02010	
MK1 and MK2	Δ*FMK1* Mel02010	
RP1 and RP2	Δ*FRP1* Mel02010	
AS1 and AS2	Δ*GAS1* Mel02010	
CK3-1	*F. oxysporum* f. sp. *lycopersici*	Imazaki et al., 2007
CK3-1-GFP	GFP-expressing CK3-1	
LF2-1	Δ*FOW2* CK3-1	Imazaki et al., 2007
LF2-1-GFP	GFP-expressing LF2-1	
MFG6	Nonpathogenic *F. oxysporum* strain	Kuroda et al., 2004
MFG6-GFP	GFP-expressing MFG6	
WPy1	*Pythium aphanidermatum*	

The oomycete *P. aphanidermatum* stain WPy1, which causes damping-off and root rot of cucurbitaceous plants was used as a melon pathogen. Strain WPy1 isolated from watermelon was kindly provided by Dr. Masaharu Kubota (National Agriculture and Food Research Organization, Japan). For preparing oospores, the strain was statically grown in V8 broth [200 mL V8 vegetable juice (Campbell’s, Camden, NJ, United States) and 3 g CaCO_3_ per liter] at 25°C for 7 days in the dark. The resulting mycelial mats were washed with sterilized water and homogenized for 30 s in a blender (Waring, New Hartford, CT, United States). Oospores were collected by filtration through cheesecloth.

DNA was extracted from *F. oxysporum* using a NucleoMag Plant Kit (Macherey-Nagel, Düren, Germany).

### Plants

Melon (*Cucumis melo* L.) cultivar Amus and tomato (*Solanum lycopersicum* L.) cultivar Ponderosa were used in inoculation tests. Seeds were sown in pots filled with fertilized granulated soil (Kumiai Nippi Engei Baido, Nihon Hiryo, Tokyo, Japan) and grown in a climate chamber at 25°C (approximately 60% relative humidity, 16 h light/8 h dark). Melon seedlings (3 weeks old) and tomato seedlings (2 weeks old) were used for inoculation tests.

### Disruption of Pathogenicity Genes

Mutants of pathogenicity-related genes *FMK1*, *FGA1*, *FGB1*, *FRP1*, and *GAS1* were generated for *F. oxysporum* (Supplementary Table 1) using transformation-mediated gene disruption. The hygromycin B resistance gene (*hph*) cassette was amplified from pSH75 by PCR using primers PtrpC_f and TtrpC_r (Supplementary Table 2). The entire exon–intron regions of pathogenicity-related genes were amplified from total DNA of strain Mel02010 by PCR using respective primer sets (Supplementary Table 2) and cloned into the plasmid pGEM-T Easy (Promega, Fitchburg, WI, United States). These plasmids were linearized by inverse PCR using primer sets that contain a 5′ overhang sequence for overlapping with sequences at the ends of the *hph* cassette (Supplementary Table 2). The linearized plasmid and the *hph* cassette were combined in the In-Fusion HD EcoDry Cloning kit (Takara Bio, Shiga, Japan) and subsequently introduced into electrocompetent *Escherichia coli* DH5α (Takara Bio). Strain Mel02010 was transformed with the resulting disruption vectors (Supplementary Table 3 and Supplementary Figure 1). Protoplasts were prepared and *F. oxysporum* was transformed as previously described (Inoue et al., 2001). Transformants were selected on regeneration media containing hygromycin B (Fujifilm-Wako Pure Chemical, Osaka, Japan) at 100 μg/mL. Disruption of the target genes in transformants were confirmed by PCR using respective primer sets (Supplementary Table 2 and Supplementary Figure 1).

### Inoculation Tests

Pathogenicity of *F. oxysporum* strains was tested by dipping roots of susceptible plants into conidial suspensions (5 × 10^5^ conidia/mL) as previously described (Inoue et al., 2001).

To evaluate the biocontrol activity of *F. oxysporum* strains, roots of melon and tomato seedlings were dipped in conidial suspensions (1 × 10^6^, 10^7^ or 10^8^ conidia/mL) of test strains for 15 s, then the seedlings were planted in pots of soil infested with conidia of the wild-type strains of *F. oxysporum* f. sp. *melonis* or f. sp. *lycopersici* (5 × 10^5^ conidia/g soil). Melon seedlings pre-inoculated with test strains were also planted in the soil infested with oospores of *P. aphanidermatum* (5 × 10^5^ oospores/g soil). Control seedlings were immersed in sterilized water and planted in infested and uninfested soils. Plants were incubated in a climate chamber at 25°C, and 16/8 h light/dark photoperiod. Disease symptoms were assessed 3 weeks after planting as follows: 0, no symptoms; 1, yellowing; 2, wilted; 3, dead.

To assess the durability of biocontrol effects after pre-inoculation of melon roots with strain Δ*FOW2* strain, pre-inoculated seedlings were grown in uninfested soil for 1, 3, or 7 days and then transplanted into soil infested with the wild-type strain (1 × 10^5^ conidia/g soil). Disease symptoms were assessed 3 weeks after planting in the infested soil.

All inoculation tests were performed at least twice to ensure reproducibility, and representative result was shown.

### Split-Root Inoculation

Root systems of melon seedlings were divided into two parts for the split-root inoculation as described previously (Larkin et al., 1996). One part was dipped in a conidial suspension of strain Δ*FOW2* (1 × 10^8^ conidia/mL) and the other in sterilized water for 15 s, then the plants were planted in a pot of soil infested with the wild-type strain (1 × 10^5^ conidia/g soil) or a pot of uninfested soil. Another part was planted in a separate pot filled with infested or uninfested soil. Plants were grown for 3 weeks, then disease symptoms were assessed as described above.

### Quantitative Real-Time PCR

Melon genes that were analyzed by qPCR are listed in Supplementary Table 4.

Roots of melon seedlings were dipped in a conidial suspension of strain Δ*FOW2* (1 × 10^8^ conidia/mL) for 15 s, and the seedlings were planted in soil infested with conidia of the wild-type strain (1 × 10^5^ conidia/g soil) or in uninfested soil. Control seedlings were immersed in sterilized water and planted in infested or uninfested soil. Three melon seedlings in each treatment were removed from soil 1, 3, or 7 days after planting. Total RNA was extracted from roots and aboveground parts using RNeasy Plant Mini Kit (Qiagen, Hilden, Germany). The qPCR of melon genes was carried out with Mx3005P QPCR System (Agilent Technologies, Santa Clara, CA, United States) using One Step SYBR PrimeScript RT-PCR Kit II (Takara Bio). Target genes encode salicylic acid-, jasmonic acid-, and ethylene-responsive resistance genes: acidic chitinase (*PR-1a*) (Uknes et al., 1992; García-Gutiérrez et al., 2013), acidic thaumatin-like protein (*PR-5a*) (Uknes et al., 1992), acetylglucosaminyltransferase (*CGT*) (Bovie et al., 2004), acidic chitinase (*PR-8*) (Metraux et al., 1989), ethylene response factor 1 (*ERF1*) (Mizuno et al., 2006) and phenylalanine ammonia-lyase gene (*PAL1*) (Diallinas and Kanellis, 1994; Supplementary Table 1). PCR primers were designed to amplify cDNA fragments of 90--150 bp on the basis of melon-expressed sequence tags in the Cucurbit Genomics Database^1^ (Supplementary Table 4). The efficiency of the primers was tested using a dilution series of genomic DNA from the melon plants. Raw data were analyzed using the 2^–ΔΔCt^ method (Livak and Schmittgen, 2001). The data were normalized to the transcript level of the actin gene, and the mRNA data of untreated melon seedlings was set to 1.0. Transcript levels of target genes in each RNA sample were measured for three independent inoculation experiments with two replicates and were analyzed for significant differences using Tukey--Kramer’s multiple range test. All statistical analyses in this study were performed with R program version 4.0.3^2^.

### Construction of Green Fluorescent Protein- and Red Fluorescent Protein-Expression Vectors and Fungal Transformation

The GFP-expression vector pTEFEGFP, in which the *eGFP* gene was fused with the *Aureobasidium pullulans TEF* promoter and the *Aspergillus awamori gla* terminator (Vanden Wymelenberg et al., 1997), was used to make *F. oxysporum* strains constitutively expressing *GFP*. The red fluorescent protein (DsRed)-expression vector pTEFDsRed was constructed as follows. The pTEFEGFP DNA was linearized by inverse PCR using primers Ptef-RFP and Tgla-RFP (Supplementary Table 2). The DsRed gene was amplified from pDsRed-Express2 (Takara Bio) by PCR using primers RFP-F and RFP-R (Supplementary Table 2). These primers contain a 5′ overhang sequence for overlapping sequences at the ends of the linearized plasmid. PCR products were combined using the In-Fusion HD EcoDry Cloning kit (Takara Bio) and subsequently introduced into electrocompetent *Escherichia coli* DH5α (Takara Bio). PCR experiments were carried out using PrimeSTAR GXL DNA Polymerase (Takara Bio) or TaKaRa Ex Taq (Takara Bio) according to the manufacturer’s instructions. The plasmids used in this study are listed in Supplementary Table 3.

The GFP-expressing vector pTEFEGFP and the DsRed-expressing vector pTEFDsRed were introduced into *F. oxysporum* strains by cotransformation with pII99, which carries the neomycin phosphotransferase gene (*nptII*) cassette (Namiki et al., 2001). Transformants carrying *nptII* cassette were selected on regeneration media containing G418 (geneticin) (Fujifilm-Wako Pure Chemical) at 200 μg/mL (Inoue et al., 2001). Hyphae of transformants were observed with a fluorescence microscope (IX73) (Olympus, Tokyo, Japan) using U-MNIB and U-MWIG filters (Olympus) to select GFP- and DsRed-expressing transformants, respectively. The selected transformants were confirmed to have hyphal growth, conidiation and pathogenicity on host plants typical of the parent strains.

### Microscopic Observations

Melon roots were pre-inoculated with conidial suspension (1 × 10^8^ conidia/mL) of the GFP-expressing strains by the root-dip method and planted in the soil infested with Mel02010-DsRed (1 × 10^5^ conidia/g soil). Three plants were removed from the soil at 3 and 7 days after planting, and conidial germination and hyphal elongation on main root surface were observed using a confocal laser scanning microscope (CLSM) (LSM-700; Carl Zeiss, Oberkochen, Germany) (GFP: excitation 488 nm and emission 509 nm; DsRed: excitation 555 nm and emission 572 nm). At least six sections of the main roots of each seedling were observed, and germination rates of conidia and hyphal lengths were measured. Data were analyzed for significant differences using Tukey–Kramer’s multiple range test.

For observing hyphae on the root surface using a scanning electron microscope, sections of the main roots were fixed twice in 2% (v/v) glutaraldehyde in 0.1 M sodium cacodylate buffer (pH 7.2) for 1 h and dehydrated using a graded ethanol series (20–100%), then immersed in 100% acetone. Samples were freeze-dried (JFD-300; JEOL, Tokyo, Japan), coated with a thin gold layer using a JEE-400 vacuum evaporator (JEOL) and observed using a JSM-5800 scanning electron microscope (JEOL).

### Selection of Thiophanate-Methyl-Resistant Strains and Inoculation Test

A conidial suspension (1 × 10^6^ cells/mL) of strain Mel02010-DsRed was irradiated for 1 to 5 min with UV light (GL15, 253.7 nm, 15W, Toshiba, Osaka, Japan) that was approximately 50 cm above the plate. The conidial suspension was plated on PDA containing 50 μg/mL thiophanate-methyl (Topsin-M; Nihon Nohyaku, Tokyo, Japan) and incubated at 25°C for 2 days. The growing colonies were subjected to single-conidial isolation three times on PDA supplemented with thiophanate-methyl, and the resistant strain Mel02010-DsRed^TR^ was selected. Strain Mel02010-DsRed^TR^ was confirmed to have normal mycelial growth, conidiation and pathogenicity to melon plants similar to the parent strain.

Melon roots were dipped in a conidial suspension (1 × 10^8^ conidia/mL) of the GFP-expressing Δ*FOW2* strain MF2-1-GFP or in sterilized water, and the seedlings were planted in soil infested with Mel02010-DsRed^TR^ (1 × 10^5^ conidia/g soil) or in uninfested soil. Soils mixed with or without thiophanate-methyl (50 μg/g soil) were used in each inoculation test. After inoculation for 24 h, seedlings were removed from three pots, and total DNA was extracted from roots using a NucleoMag Plant Kit (Macherey-Nagel). Fungal DNA was detected from root DNA by PCR amplification of the GFP gene of MF2-1-GFP and the DsRed gene of Mel02010-DsRed^TR^ using Takara Ex Taq (Takara) and specific primers for each gene (Supplementary Table 2). The elongation factor gene (*EF1*α) of melon was also amplified as a standard. The seedlings were grown for 3 weeks, and disease symptoms were assessed as described above.

### Carbon Source Competition Assay

Carbon source competition in soil between nonpathogenic and wild-type strains was assayed using the buried membrane filter method of Larkin and Fravel (1999) with a slight modification. Fertilized granulated soil (Kumiai Nippi Engei Baido) was mixed with a glucose solution at final concentrations from 0 to 0.4 mg/g soil. One milliliter of 3 × 10^4^ conidia/mL of the wild-type strain Mel02010 was deposited on a cellulose membrane filter (MF-Millipore, 0.45-μm pore, 47 mm diameter; Millipore, Billerica, MA, United States) by vacuum filtration. The membrane filters were buried in soil infested with nonpathogenic strains (1 × 10^5^ conidia/g soil) and incubated in a moist chamber at 25°C for 3 days in the dark. The filters were removed from soil, rinsed with sterilized water and boiled in lactophenol-aniline blue solution (0.01% aniline blue in lactic acid/phenol/dH_2_O/glycerol [1:1:1:1, v/v/v/v]). Length of hyphae elongated from conidia on the filters was measured using a light microscope (IX73, Olympus). At least 100 germinated conidia were observed in each sample, and the mean lengths were compared for significant differences among treatments using Student’s *t*-test.

## Results

### Biocontrol Activities of Pathogenicity Mutants Against the Wild-Type Strain

Imazaki et al. (2007) isolated Δ*FOW2* strains from strain Mel02010 of *F. oxysporum* f. sp. *melonis* and found that the mutants were not pathogenic to host plants. Here, we generated mutants of five other known pathogenicity genes from strain Mel02010: *FMK1* encoding protein kinase (Di Pietro et al., 2001), *FGA1* and *FGB1* encoding G protein subunit α and β, respectively (Jain et al., 2002; 2003), *FRP1* encoding an F-box protein (Duyvesteijn et al., 2005) and *GAS1* encoding a putative β-1,3-glucanosyltransferase (Caracuel et al., 2005; Supplementary Table 1). To generate mutants of these genes, we used homologous recombination to replace each gene with the plasmid, which contains the target gene fragment interrupted with the *hph* cassette (Supplementary Figure 1). Two independent mutants of each gene were assayed for pathogenicity on melon plants. Mutation of *FMK1* and *FRP1* in strain Mel02010 caused a drastic reduction in pathogenicity (Supplementary Figure 2). Although mutations of *FGA1*, *FGB1*, and *GAS1* reduced virulence, some of the inoculated plants still died or were severely wilted within 21 days post inoculation (dpi) (Supplementary Figure 2). The Δ*FOW2* strains were confirmed to lack pathogenicity on melon plants (Imazaki et al., 2007).

**FIGURE 1:**
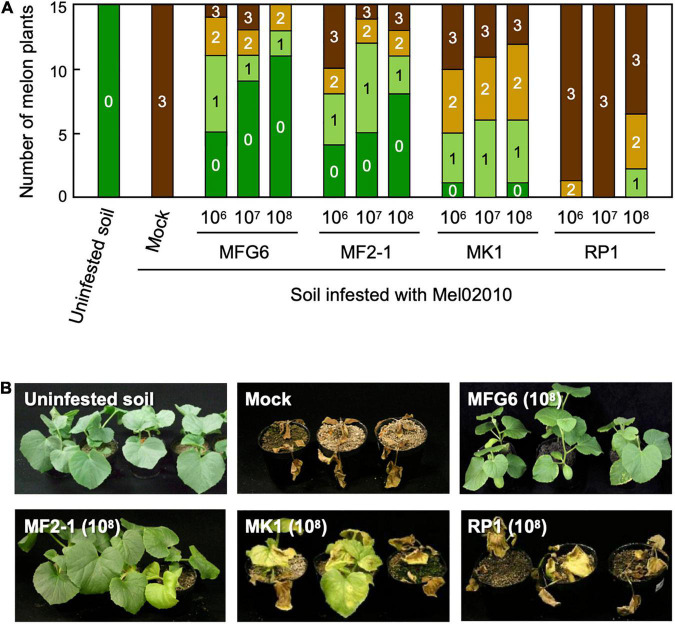
Biocontrol activity of pathogenicity gene mutants of *Fusarium oxysporum* f. sp. *melonis* against the parental strain. Roots of melon seedlings were dipped in a conidial suspension (1 × 10^6^, 10^7^ or 10^8^ conidia/mL) of Δ*FOW2* (MF2-1), Δ*FMK1* (MK1), and Δ*FRP1* (RP1) strains of *F. oxysporum* f. sp. *melonis* or nonpathogenic strain MFG6. The seedlings were planted in soil infested with the parental wild-type strain Mel02010 (1 × 10^5^ conidia/g soil). Control seedlings were immersed in water and planted in uninfested or infested soil (Mock). **(A)** Symptoms were scored at 21 dpi as 0, no symptoms; 1, yellowing; 2, wilted; 3, dead. **(B)** Plants at 21 dpi.

We tested for biocontrol activity of Δ*FMK1*, Δ*FRP1*, and Δ*FOW2* strains against the wild-type strain Mel02010. Melon seedlings were pre-inoculated with a conidial suspension (1 × 10^6^, 10^7^ or 10^8^ conidia/mL) of each mutant by the root-dip method and grown in soil infested with Mel02010 (10^5^ conidia/g soil). The nonpathogenic *F. oxysporum* strain MFG6, which was previously identified to have biocontrol activity against *F. oxysporum* pathogens, was also used for pre-inoculation. Roots of control seedlings were dipped in water and planted in infested soil, and within 15 dpi, plants had yellowing and wilting, and all plants died within 21 dpi (Figure 1). However, seedlings pre-inoculated with Δ*FOW2* strain MF2-1 or MFG6 were markedly reduced in disease severity, and some had no symptoms (Figure 1). The level of biocontrol activity was dependent on the concentration of conidia used for the pre-inoculations and was similar for MF2-1 and MFG6 (Figure 1). Such results were reproduced using another Δ*FOW2* strain MF2-2 (Supplementary Figure 3B). Symptom development on seedlings pre-inoculated with Δ*FMK1* strain MK1 was delayed, and severity was significantly reduced (Figure 1). Seedlings pre-inoculated with Δ*FRP1* strain RP1 had slightly lower disease severity (Figure 1). These results suggest that the weaker the virulence, the higher the biocontrol activity.

### Biocontrol Activities of Δ*FOW2* Strains

**FIGURE 2:**
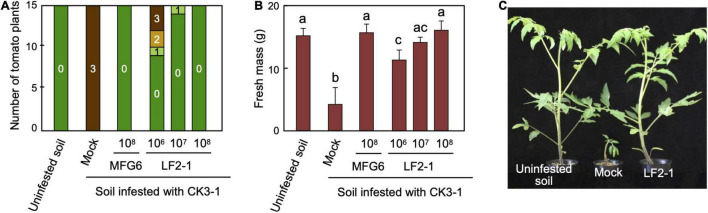
Biocontrol activity of pathogenicity gene mutants of *Fusarium oxysporum* f. sp. *lycopersici* against the parental strain. Roots of tomato seedlings were dipped in conidial suspensions (1 × 10^6^, 10^7^ or 10^8^ conidia/mL) of Δ*FOW2* strain LF2-1 of *F. oxysporum* f. sp. *lycopersici* or nonpathogenic strain MFG6. The seedlings were planted in soil infested with parental wild-type strain CK3-1 (1 × 10^5^ conidia/g soil). Control seedlings were immersed in water and planted in uninfested or infested soil (Mock). **(A)** Symptoms were scored 21 dpi as 0, no symptoms; 1, yellowing; 2, wilted; 3, dead. **(B)** Fresh mass of aboveground parts of tomato seedlings (*n* = 15) at 21 dpi. Data represent averages and standard errors based on 15 seedlings. Columns with different letters indicate that means differed significantly at *P* ≤ 0.01 in the Tukey–Kramer multiple range test. **(C)** Plants at 21 dpi.

*FOW2* is conserved in *F. oxysporum* pathogens that infect different plants and is also essential for pathogenicity of *F. oxysporum* f. sp. *lycopersici* on tomato plants (Imazaki et al., 2007). We tested biocontrol activity of the Δ*FOW2* strain LF2-1 generated from *F. oxysporum* f. sp. *lycopersici*. Tomato seedlings were pre-inoculated with conidial suspensions (1 × 10^6^, 10^7^ or 10^8^ conidia/mL) of LF2-1 and planted in soil infested with the wild-type strain CK3-1 (10^5^ conidia/g soil). Mock seedlings in the infested soil died within 21 dpi (Figure 2). Pre-inoculation of tomato roots with a conidial suspension of LF2-1 apparently reduced disease severity in a concentration-dependent manner (Figure 2).

**FIGURE 3:**
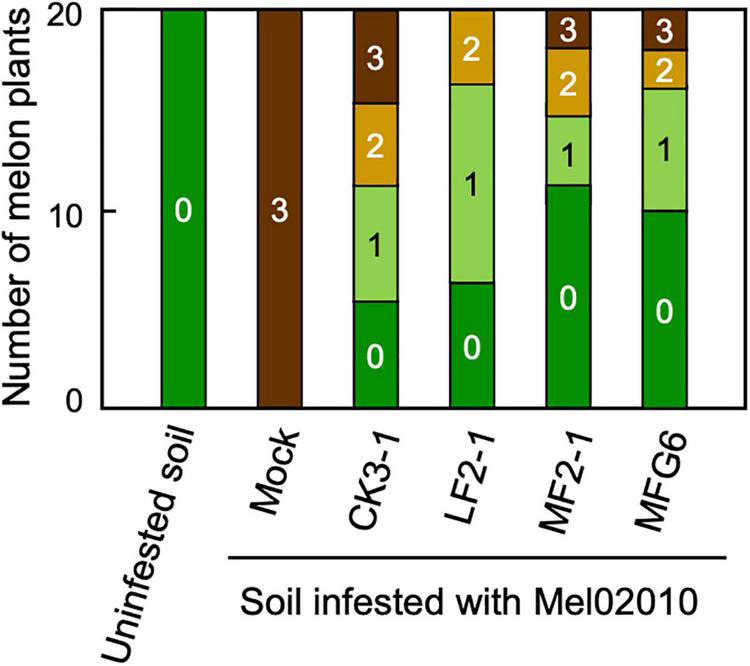
Biocontrol activity of *Fusarium oxysporum* f. sp. *lycopersici* and its mutant Δ*FOW2* strain against *F. oxysporum* f. sp. *melonis*. Roots of melon seedlings were dipped in a conidial suspension (1 × 10^8^ conidia/mL) of *F. oxysporum* f. sp. *lycopersici* strain CK3-1 or Δ*FOW2* strain LF2-1, and the seedlings were planted in soil infested with *F. oxysporum* f. sp. *melonis* strain Mel02010 (1 × 10^5^ conidia/g soil). Control seedlings were immersed in water and planted in uninfested or infested soil (Mock). Symptoms were scored at 21 dpi as 0, no symptoms; 1, yellowing; 2, wilted; 3, dead. MF2-1, Δ*FOW2* strain of *F. oxysporum* f. sp. *melonis*; MFG6, nonpathogenic *F. oxysporum*.

We also tested the biocontrol activity of *F. oxysporum* f. sp. *lycopersici* against *F. oxysporum* f. sp. *melonis* on melon plants. Melon seedlings pre-inoculated with wild-type or Δ*FOW2* strain of *F. oxysporum* f. sp. *lycopersici* had significantly reduced disease severity (Figure 3), indicating that all nonpathogenic strains used were active against *F. oxysporum* f. sp. *melonis*. These results suggest that the biocontrol activity of Δ*FOW2* strains would be due to their nonpathogenic nature, but not to the *FOW2* disfunction itself.

**FIGURE 4:**
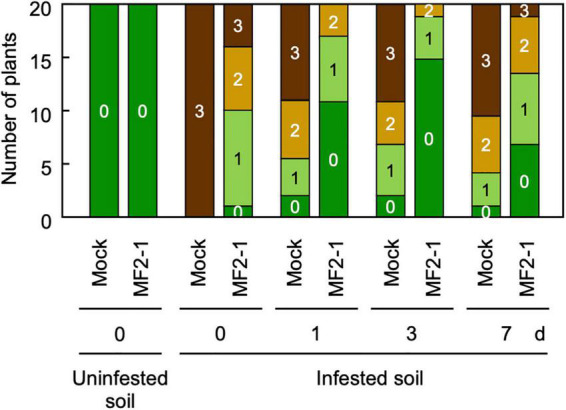
Persistence of biocontrol effect after pre-inoculation of melon seedlings with Δ*FOW2* strain. Roots of melon seedlings were dipped in a conidial suspension (1 × 10^8^ conidia/mL) of Δ*FOW2* strain MF2-1 or in water, and seedlings were planted in soil infested with wild-type strain Mel02010 (1 × 10^5^ conidia/g soil) or in uninfested soil. After 1, 3, or 7 days, seedlings grown in uninfested soil were transplanted in Mel02010 infested soil. Symptoms were scored 3 weeks after planting in infested soil as 0, no symptoms; 1, yellowing; 2, wilted; 3, dead.

We examined the durability of the biocontrol effect after melon roots were pre-inoculated with Δ*FOW2* strain MF2-1, planted in uninfested soil, then transplanted after 1, 3, and 7 days in soil infested with Mel02010 conidia. Mock seedlings without MF2-1 pre-inoculation developed severe symptoms within 21 dpi (Figure 4), whereas symptoms on MF2-1-pre-inoculated seedlings were less severe, and the transplanting timing did not conspicuously affect the biocontrol activity (Figure 4). These results indicated that the biocontrol effect lasted for at least 7 days.

### Effect of Pre-inoculation of Melon Roots With Δ*FOW2* Strain on Expression of Defense-Related Genes

**FIGURE 5:**
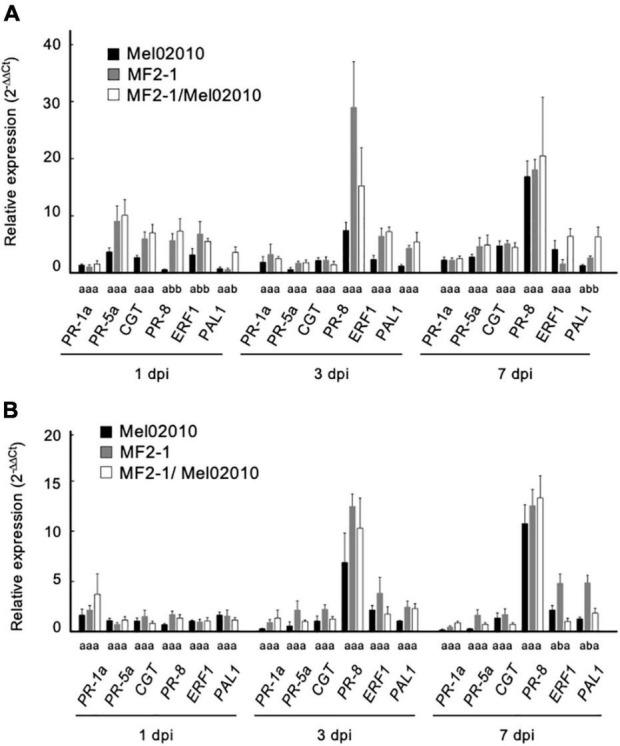
Transcription levels of defense-related genes in melon seedlings inoculated with Δ*FOW2* strain. Roots of melon seedlings were dipped in a conidial suspension (1 × 10^8^ conidia/mL) of Δ*FOW2* strain MF2-1, and seedlings were planted in soil uninfested (MF2-1) or infested with the wild-type strain Mel02010 (1 × 10^5^ conidia/g soil) (MF2-1/Mel02010). Control seedlings were immersed in water and planted in Mel02010 infested soil (Mel02010) or uninfested soil. Seedlings were removed from soil 1, 3, or 7 dpi, and total RNA was extracted from roots **(A)** and aboveground parts **(B)** for quantitative real-time PCR analyses. Raw data were analyzed using the 2^–ΔΔCt^ method. Data were normalized against the actin gene, and those of uninoculated melon seedlings was set to 1.0. Error bars indicate the standard errors calculated from three independent inoculation experiments with two replicates. Columns with different letters indicate that means differed significantly at *P* ≤ 0.01 in the Tukey–Kramer multiple range test.

The transcription levels of salicylic acid-, jasmonic acid-, and ethylene-responsive resistance genes in roots and aboveground part of inoculated plants were evaluated using qPCR and were compared among plants different in treatments: plants grown in Mel02010 infested soil without pre-inoculation, plants pre-inoculated with Δ*FOW2* strain MF2-1 and grown in uninfested soil; plants pre-inoculated with MF2-1 and grown in Mel02010 infested soil. Although all the target genes except *PR-1a* tended to be upregulated by MF2-1 pre-inoculation in roots and aboveground parts, statistically significant differences were detected at 1 and 7 dpi only for *PR-8*, *ERF1*, and *PAL1* in plants pre-inoculated with MF2-1 (Figure 5). These results suggest that the Δ*FOW2* strain did not markedly induce persistent expression of defense-related genes and that the biocontrol activity of the Δ*FOW2* strain was not due primarily to the induction of resistance, since these expression patterns were not consistent with the durability of the biocontrol effect lasting for 7 days.

To assess the systemic resistance induced by the pre-inoculation of melon seedlings with the Δ*FOW2* strain, we used a split-root inoculation method. Symptom development on the seedlings was similar to that on those without MF2-1, indicating that systemic resistance was not induced by the inoculation of roots with the Δ*FOW2* strain (Supplementary Figure 4).

### Colonization of Wild-Type Strain on Melon Roots Pre-inoculated With Δ*FOW2* Strain

We observed the effect of pre-inoculation of melon roots with the Δ*FOW2* strain on colonization of the wild-type strain on root surface. Melon seedlings were inoculated with the GFP-expressing Δ*FOW2* strain MF2-1-GFP and planted in the soil infested with the thiophanate-methyl-tolerant wild-type strain Mel02010-DsRed^TR^. From total DNA of roots grown in thiophanate-methyl mixed soils, PCR amplified the DsRed gene fragments, but not the GFP gene fragments, confirming that MF2-1-GFP was not viable in the soil (Supplementary Figure 5). Although MF2-1-GFP had remarkable biocontrol activity against Mel02010-DsRed^TR^ in the absence of thiophanate-methyl in the soil, it lost its activity when thiophanate-methyl was present (Supplementary Figure 5), indicating that root colonization by the Δ*FOW2* strain was important for the biocontrol effect.

**FIGURE 6:**
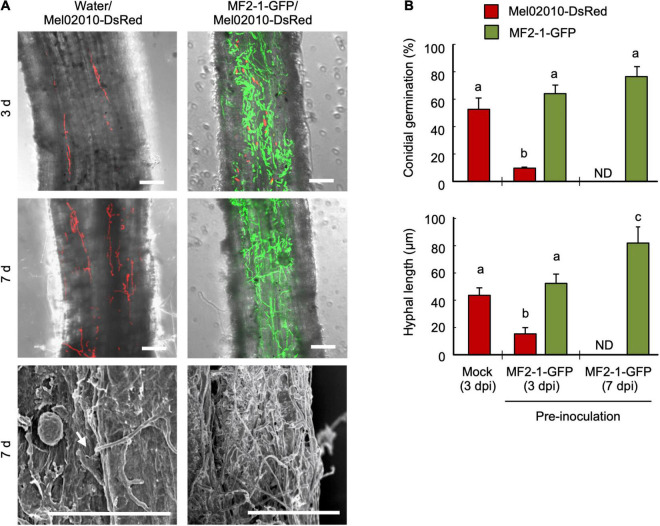
Inhibition of conidial germination and hyphal growth of wild-type strain on root surface after pre-inoculation with Δ*FOW2* strain. Roots of melon seedlings were dipped in a conidial suspension (1 × 10^8^ conidia/mL) of Δ*FOW2* strain MF2-1-GFP or in water, and seedlings were planted in soil infested with wild-type strain Mel02010-DsRed (1 × 10^5^ conidia/g soil). Seedlings were removed from soil at 3 and 7 dpi. **(A)** The root surface was viewed with laser scanning confocal microscopy (LSCM; top and middle) or scanning electron microscopy (bottom). Bar = 50 μm. Arrow: possible penetration site. **(B)** Percentage of conidial germination and hyphal length of each strain on main roots at 3 and 7 dpi was determined using LSCM. Data represent means and standard errors of three replications. Columns with different letters indicate that means differed significantly at *P* ≤ 0.01 in the Tukey–Kramer multiple range test. ND, not detected.

After roots of melon seedlings were dipped in a conidial suspension of MF2-1-GFP or in water, then planted in Mel02010-DsRed infested soil, CLSM showed that conidia of Mel02010-DsRed on the roots dipped in water had germinated and that hyphae had elongated by 3 dpi and, by 7 dpi, penetration hyphae were frequently observed in the epidermal cells (Figure 6 and Supplementary Figure 6). On roots pre-inoculated with MF2-1-GFP, conidia of MF2-1-GFP had germinated and hyphae had elongated extensively on the root surface at 3 dpi (Figure 6A). In contrast, hyphae of Mel02010-DsRed were occasionally observed (Figure 6A). At 7 dpi, MF2-1-GFP had formed an extensive hyphal network that covered almost the entire root surface, and fluorescence from Mel02010-DsRed was not detected (Figure 6B). Conidial germination rates and hyphal lengths of Mel02010-DsRed and MF2-1-GFP on root surfaces at 3 and 7 dpi showed that germination and hyphal elongation of Mel02010-DsRed were inhibited when melon roots were pretreated with MF2-1-GFP.

**FIGURE 7:**
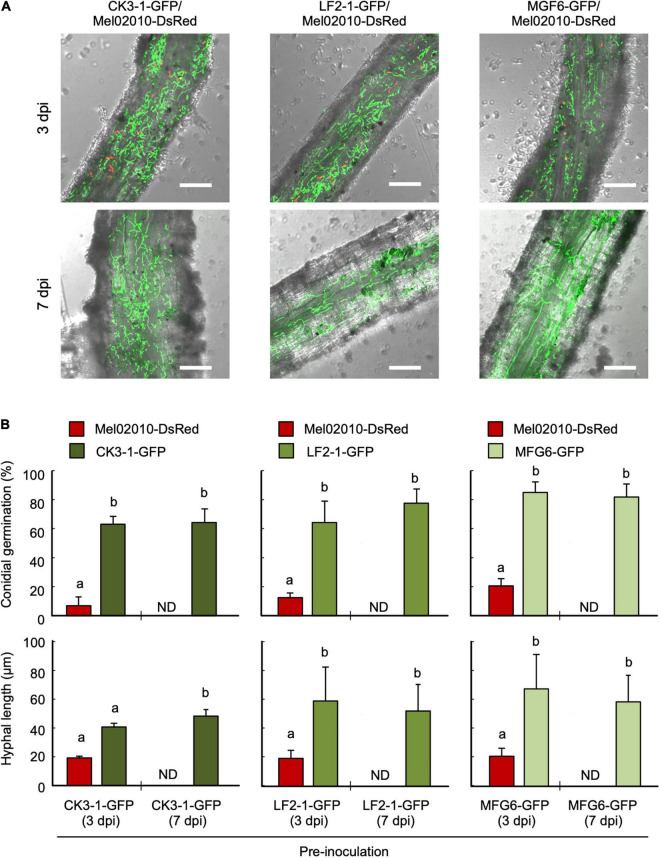
Inhibition of conidial germination and hyphal growth of *F. oxysporum* f. sp. *melonis* on root surface by the pre-inoculation of *F. oxysporum* f. sp. *lycopersici* and its Δ*FOW2* strain. Roots of melon seedlings were dipped in a conidial suspension (1 × 10^8^ conidia/mL) of *F. oxysporum* f. sp. *lycopersici* strains or in water, and seedlings were planted in soil infested with *F. oxysporum* f. sp. *melonis* strain (1 × 10^5^ conidia/g soil). Seedlings were removed from soil at 3 and 7 dpi. **(A)** Root surface viewed with a laser scanning confocal microscopy (LSCM). Bar = 50 μm. **(B)** Percentage of conidial germination and hyphal length of each strain on main roots was determined 3 and 7 dpi using LSCM. Data represent means and standard errors of three replications. Columns with different letters indicate that means differed significantly at *P* ≤ 0.01 in the Tukey–Kramer multiple range test. ND, not detected. Mel02010-DsRed, DsRed-expressing strain of *F. oxysporum* f. sp. *melonis*; CK3-1-GFP, GFP-expressing strain of *F. oxysporum* f. sp. *lycopersici*; LF2-1-GFP, *FOW2* mutant of CK3-1-GFP; MGF6-GFP, GFP-expressing nonpathogenic *F. oxysporum* strain.

Melon seedlings were pre-inoculated with conidial suspensions of strain CK3-1-GFP and its Δ*FOW2* strain LF2-1-GFP of *F. oxysporum* f. sp. *lycopersici* and nonpathogenic strain MFG6-GFP by the root-dip method and planted in the soil infested with Mel02010-DsRed. Conidia of pre-inoculated strains germinated, and hyphae had elongated extensively on the root surface similar to MF2-1-GFP, whereas conidial germination and hyphal elongation of Mel02010-DsRed on the root surface were significantly inhibited (Figure 7).

As seen with CLSM of MF2-1-GFP pre-inoculated roots, hyphae had not colonized epidermal cells by 7 dpi (Supplementary Figure 6), confirming that the Δ*FOW2* strain could not penetrate and colonize the epidermal cells; it only colonized the root surface. Strains CK3-1-GFP, LF2-1-GFP, and MFG6-GFP also colonized the melon root surface, but not in the epidermal cells (Supplementary Figure 6). These results strongly suggest that pre-colonization of the root surface by nonpathogenic strains inhibits conidial germination and hyphal development of the soil-borne, wild-type strain.

### Carbon Source Competition Between Wild-Type and Δ*FOW2* Strain in the Rhizosphere

**FIGURE 8:**
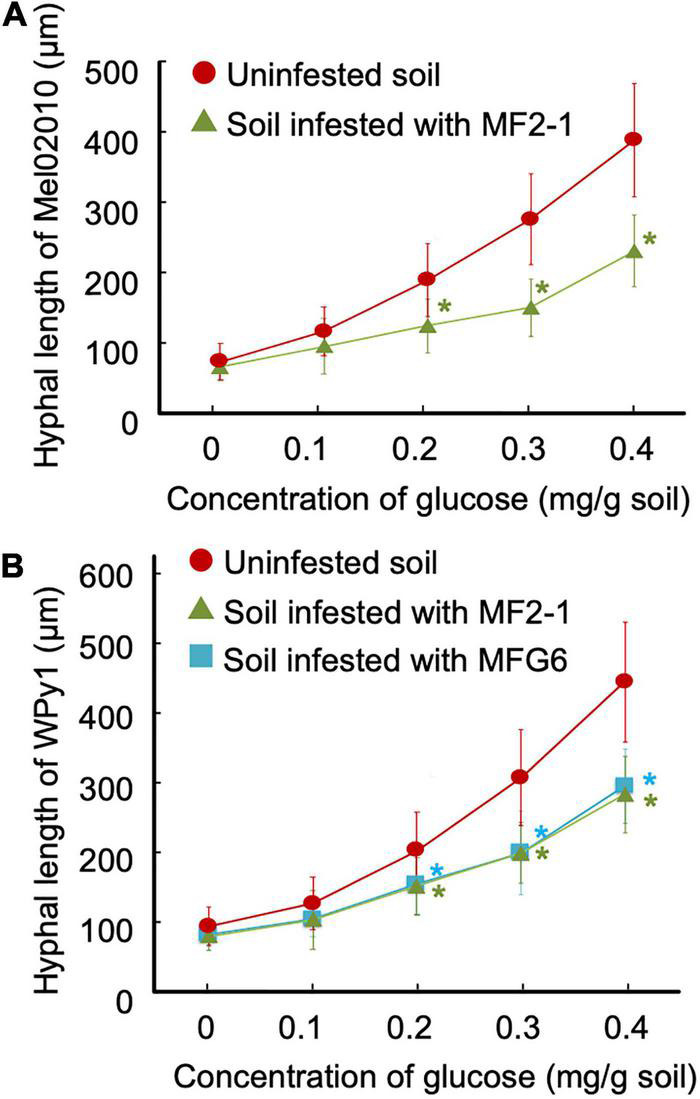
Carbon source competition between pathogenic and nonpathogenic strains in soil. Cellulose membrane filters with conidia of strain Mel02010 of *Fusarium oxysporum* f. sp. *melonis* **(A)** and oospores of strain WPy1 of *Pythium aphanidermatum* **(B)** were set in soil mixed with glucose solution (0–0.4 mg/g soil) and a conidial suspension (1 × 10^5^ conidia/g soil) of nonpathogenic strains. After 3 days, the filters were removed from the soil, and the length of hyphae of Mel02010 and WPy1 was measured. Data represent means and standard errors of three replicates. Asterisks indicate a significant difference in hyphal length from that in uninfested soil at each glucose concentration (*P* < 0.001; Student’s *t*-test). MF2-1, *FOW2* mutant of Mel02010; MFG6, nonpathogenic *F. oxysporum*.

Conidial germination and hyphal elongation of the wild-type strain were markedly inhibited on the root surface pre-inoculated with the Δ*FOW2* strain (Figure 6). The Δ*FOW2* strain, however, did not inhibit conidial germination or hyphal elongation of the wild-type strain on any artificial media (data not shown). In the verification of carbon source competition using cellulose membrane filters with conidia of the wild-type strain Mel02010 set in soil mixed with glucose solution (0–0.4 mg/g soil) and conidia (1 × 10^5^ conidia/g soil) of the Δ*FOW2* strain MF2-1 (Supplementary Figure 7A), after 3 days, mean hyphal length of Mel02010 had increased proportionally to glucose concentrations and was significantly reduced in the presence of MF2-1 conidia in the soil (Figure 8A). Hyphal elongation of Mel02010 was also inhibited in the presence of strain CK3-1 of *F. oxysporum* f. sp. *lycopersici*, its Δ*FOW2* strain LF2-1 or nonpathogenic MFG6 in the soils (Supplementary Figure 7B). Pre-inoculation of melon roots with these strains also inhibited conidial germination and hyphal elongation of Mel02010 on melon roots (Figures 6, 7). These results suggest that these strains pre-colonized on the root surface have a competitive advantage over Mel02010 for carbon sources in the rhizosphere.

### Biocontrol Activities of Δ*FOW2* Strain Against *Pythium aphanidermatum*

**FIGURE 9:**
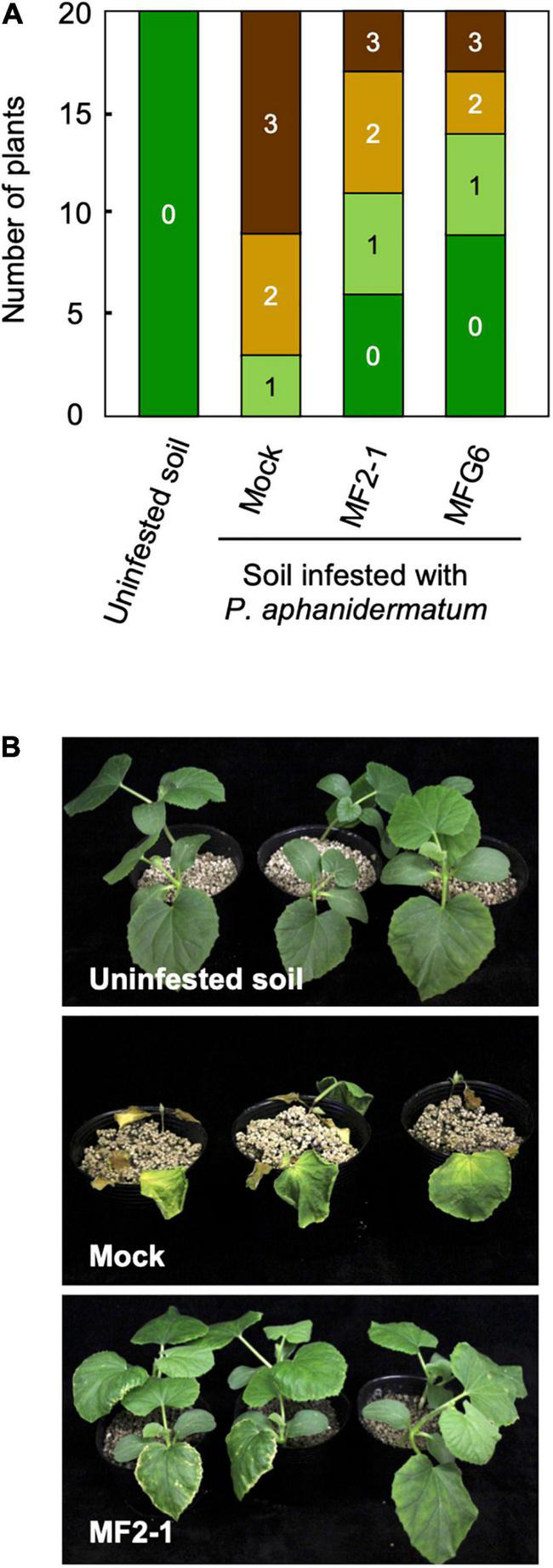
Biocontrol activity of Δ*FOW2* strain of *Fusarium oxysporum* f. sp. *melonis* against *Pythium aphanidermatum*. Roots of melon seedlings were dipped in a conidial suspension (1 × 10^6^, 10^7^ or 10^8^ conidia/mL) of Δ*FOW2* strain MF2-1 or nonpathogenic strain MFG6, and seedlings were planted in soil infested with *P. aphanidermatum* (1 × 10^5^ oospores/g soil). Control seedlings were immersed in water and planted in uninfested or infested soil (Mock). **(A)** Symptoms were scored 21 dpi as 0, no symptoms; 1, yellowing; 2, wilted; 3, dead. **(B)** Plants at 21 dpi.

In the test of the biocontrol activity of the Δ*FOW2* strain MF2-1 against *P. aphanidermatum*, melon seedlings root-dipped in 1 × 10^8^ conidia/mL of strain MF2-1 or nonpathogenic strain MFG6 and planted in soil infested with *P. aphanidermatum* strain WPy1 (10^5^ oospores/g soil). Mock seedlings without pre-inoculation showed yellowing and damping-off by 14 dpi, and more than half of the seedlings had died by 21 dpi (Figure 9A). MF2-1-pre-inoculated plants in infested soil had chlorotic leaf edges, but symptoms were less severe, and pre-inoculation of seedlings with MFG6 also reduced severity (Figure 9B).

We also verified the carbon source competition between *P. aphanidermatum* and *F. oxysporum* strains in soil using oospores of strain WPy1 on cellulose membrane filters and set in the soil mixed with glucose solution (0–0.4 mg/g soil) and conidia (1 × 10^5^ conidia/g soil) of MF2-1 or MFG6. After 3 days, hyphal growth of WPy1 was significantly inhibited in soils mixed with conidia of MF2-1 and MFG6 (Figure 8B). These results indicate that *F. oxysporum* strains also protect melon plants from infection with *P. aphanidermatum* probably due to their pre-colonization on the root surface.

## Discussion

In tests of three strains with a mutation in one of three pathogenicity genes that are conserved in *F. oxysporum* for activity against a pathogenic strain of the fungus, pre-inoculation with the Δ*FOW2* strain yielded the best biocontrol against pathogenic strains of *F. oxysporum* ff. spp. *melonis* and *lycopersici*, and its activity was concentration dependent, like that of the biocontrol by nonpathogenic *F. oxysporum* strain MFG6. The same level of biocontrol was maintained against the wild-type strain for at least 7 days after pre-inoculation (Figure 4). Since the wild-type strain has already penetrated the root by 7 dpi when the biocontrol agent is not present (Supplementary Figure 6), we tested whether Δ*FOW2* strain had induced a plant defense response by 7 days. The qPCR analysis revealed only limited expression levels of some ethylene (*ERF1*) or jasmonic acid-responsive (*PAL1*) resistance genes in roots after pretreatment with strain Δ*FOW2* (Figure 5A), which did not correlate with the 7-day biocontrol effect of the Δ*FOW2* strain. None of the defense genes were expressed in the systemic aboveground parts of plants inoculated with the Δ*FOW2* strain and the wild-type strains (Figure 5B). Likewise, defense-related genes in tomato that were highly induced by a chitin synthase-deficient strain that was less virulent than the parental strain of *F. oxysporum* f. sp. *lycopersici* (Pareja-Jaime et al., 2010), were not distinctly altered in their expression in the roots or aboveground parts of tomato pre-inoculated with the Δ*FOW2* strain (data not shown). No biocontrol effect was observed in the systemically infected roots (Supplementary Figure 4). These results suggest that the high biocontrol activity of the Δ*FOW2* strain is not primarily due to the induction of plant resistance.

We then analyzed the effect of colonization by the Δ*FOW2* strain on the infection process of the wild-type strain, which is tolerant to the chemical fungicide thiophanate-methyl. The Δ*FOW2* strain was eradicated by thiophanate-methyl before its conidia germinated and hyphae grew, and the wild-type strain infected the host plants (Supplementary Figure 5), suggesting that preemptive colonization of Δ*FOW2* is essential for the biocontrol. Interestingly, conidial germination and hyphal elongation were inhibited only in the wild-type strain on the root pre-inoculated with the Δ*FOW2* strain, and the DsRed fluorescence of the wild-type strain had disappeared from the root surface by 3 dpi (Figure 6). Nevertheless, the pathogenic strain was still viable in the rhizosphere soil. In addition, the Δ*FOW2* strain colonizes the roots more rapidly than the wild-type strain does, even though their growth rates do not differ in culture (Imazaki et al., 2007). The Δ*FOW2* strain was not detected from inside the roots, meaning that it grows epiphytically on the root surface. This characteristic hyphal growth on melon roots was the same as that of *F. oxysporum* f. sp. *lycopersici* CK3-1 and nonpathogenic strain MFG6 when they were used for pre-inoculation (Figure 7). These results suggest that pre-inoculation with the Δ*FOW2* strain reduces the proliferation of wild-type strains on the plant surface, probably because the extensive hyphal network of the Δ*FOW2* strain covers the limited number of infection sites before the wild-type strain can reach them or a resistance mechanism is triggered that was not detected by any marker genes that we used.

In fact, it has been proposed that nonpathogenic biocontrol strains of *F. oxysporum* might induce a novel resistance pathway that differs from the well-studied plant hormone-mediated signaling pathways and microbe-associated molecular patterns (MAMPs)-triggered immunity: endophytic *F. oxysporum* strain Fo47 does not affect expression of salicylic acid, jasmonic acid, and ethylene responsive marker genes and is still able to control Fusarium wilt in tomato plants that are impaired in these plant hormones (Constantin et al., 2019). Nor is MAMPs-triggered immunity involved in Fo47-mediated resistance (de Lamo et al., 2020). However, this hypothesized pathway is unlikely to be involved in the biocontrol activity of the Δ*FOW2* strain. Since Δ*FOW2* strain is genetically identical to its parental strain *F. oxysporum* f. sp. *melonis*, the high selectivity of the biocontrol that appears only against pathogenic *F. oxysporum* cannot be explained by plant-mediated resistance alone. Similar to our findings in this study, the biocontrol activity of nonpathogenic *F. oxysporum* eliminates only pathogenic *F. oxysporum* from the root surface and has no effect on the hyphal growth of nonpathogenic *F. oxysporum* (Olivain et al., 2003; Bolwerk et al., 2005). This high selectivity of biocontrol that appears commonly only against pathogenic *F. oxysporum* may be more strongly related to direct interaction with the nonpathogenic strains of *F. oxysporum*. For example, the sesquiterpene volatile α-humulenone emitted by nonpathogenic *F. oxysporum* strain MSA35 represses the expression of pathogenicity genes in *F. oxysporum* (Minerdi et al., 2009). The endophyte *Serendipita indica* suppresses virulence of the barley root pathogen *Bipolaris sorokiniana* by affecting expression of genes involved in secondary metabolism and effectors (Sarkar et al., 2019). Trophic interactions might also explain the mechanisms of biocontrol by the Δ*FOW2* strain because the mutation in the *FOW2* gene does not affect the ability of the mutant to use any amino acids, nitrogen, or carbon sources (Imazaki et al., 2007).

In carbon source competition assays of nonpathogenic *F. oxysporum* strains that are well-known biocontrol agents, strain Fo47 competes for carbon source against pathogenic *F. oxysporum*, but strain CS-20, which mainly induces plant resistance, does not compete for glucose (Larkin and Fravel, 1999). Here, we found that not only the Δ*FOW2* strains (MF2-1 and LF2-1), but also the pathogenic strain of *F. oxysporum* f. sp. *lycopersici* competed with *F. oxysporum* f. sp. *melonis* for a carbon source in the soil. Thus, the mutation in the *FOW2* gene is not relevant to nutrient competition. Also, the Δ*FOW2* strain protected melon plants from the root rot by the oomycete *P. aphanidermatum* and competed for glucose in the soil. High population densities give nonpathogenic *F. oxysporum* strains an advantage in competition for the carbon sources against pathogens in the rhizosphere. The inoculum density of the endophytic strain Fo47 must be much higher than that of the pathogenic strain since it confers protection primarily through competition for nutrients and niches (Alabouvette et al., 1993; Lemanceau et al., 1993; Larkin and Fravel, 1999; Fravel et al., 2003; Bolwerk et al., 2005). In oligotrophic environments such as soil, nutrient competition between microorganisms is frequent, leading to soil fungistasis—the inhibition of conidial germination and/or hyphal growth—as observed in this study (Lockwood, 1977; de Boer et al., 2003).

Pre-inoculation with an avirulent strain of *F. oxysporum*, even a pathogenic strain belonging to another forma specialis, results in the mitigation of symptoms due to the mechanism called cross protection (Matta, 1989; Silvar et al., 2009). In the present study, *F. oxysporum* f. sp. *lycopersici* was highly effective against a virulent strain of *F. oxysporum* f. sp. *melonis* on melon and behaved similarly to the Δ*FOW2* strains on the root surface. The Δ*FOW2* strains of *F. oxysporum* f. sp. *lycopersici* had a protective effect similar to its parental strain on non-host melon in pre-inoculation tests (Figure 2), suggesting that the biocontrol effect of the Δ*FOW2* strains would not be due to the mutation of *FOW2* gene, but rather to cross protection based on its nonpathogenic nature. Likewise, a nonpathogenic mutant of the transcriptional regulator gene *SGE1*, which is required for penetration of the root cortex and expression of genes encoding effectors, and its parental strain *F. oxysporum* f. sp. *lycopersici* have a similar protective effect on the non-host flax plant against the flax pathogen *F. oxysporum* f. sp. *lini* (Michielse et al., 2009).

In summary, to elucidate the molecular basis for biocontrol by nonpathogenic *F. oxysporum* against the wilt disease caused by pathogenic *F. oxysporum*, we assessed the biocontrol activities of nonpathogenic mutants generated by disruption of pathogenicity genes. The Δ*FOW2* strain strains had concentration-dependent biocontrol activity against root-borne pathogens *F. oxysporum* and *P. aphanidermatum*. The Δ*FOW2* strains and the nonpathogenic strains such as MFG6 and *F. oxysporum* f. sp. *lycopersici* colonized the melon root surface and inhibited conidial germination and hyphal elongation of pathogenic *F. oxysporum*, probably by outcompeting for nutrients rather than by inducing resistance. Nonpathogenic strains, including Δ*FOW2* strains, never compete against themselves for nutrients, suggesting that nonpathogenic strains can distinguish themselves from pathogenic strains. Since nutrient competition occurs only against strains that are pathogenic to plants, nonpathogenic strains might recognize pathogenic strains via virulence factors such as effector proteins or secondary metabolites secreted from pathogenic strains.

## Data Availability Statement

The original contributions presented in the study are included in the article/Supplementary Material, further inquiries can be directed to the corresponding author.

## Author Contributions

YI and TT conceived and designed the experiments. YI, AO, HS, and YH carried out fungal transformation and inoculation tests. YI, HK, and YH performed qPCR analysis. YI and ON observed by laser scanning confocal microscopy and scanning electron microscopy. YI and TT analyzed all data, prepared tables, figures, and additional materials, and wrote the manuscript. All authors contributed to the article and approved the submitted version.

## Conflict of Interest

The authors declare that the research was conducted in the absence of any commercial or financial relationships that could be construed as a potential conflict of interest.

## Publisher’s Note

All claims expressed in this article are solely those of the authors and do not necessarily represent those of their affiliated organizations, or those of the publisher, the editors and the reviewers. Any product that may be evaluated in this article, or claim that may be made by its manufacturer, is not guaranteed or endorsed by the publisher.

## Footnotes

1
http://www.icugi.org/

2
www.r-project.org

## References

[B1] Alabouvette C. (1986). Fusarium-wilt suppressive soils from the chateaurenard region - review of a 10-year study. *Agronomie* 6 273–284. 10.1051/agro:19860307

[B2] Alabouvette C. Lemanceau P. Steinberg C. (1993). Recent advances in biological control of fusarium wilts. *Pestic. Sci.* 37 365–373. 10.1002/ps.2780370409

[B3] Alabouvette C. Olivain C. Migheli Q. Steinberg C. (2009). Microbiological control of soil-borne phytopathogenic fungi with special emphasis on wilt-inducing *Fusarium oxysporum*. *New Phytol.* 184 529–544. 10.1111/j.1469-8137.2009.03014.x 19761494

[B4] Bolwerk A. Lagopodi A. L. Lugtenberg B. J. J. Bloemberg G. V. (2005). Visualization of interactions between a pathogenic and a beneficial *Fusarium* strain during biocontrol of tomato foot and root rot. *Mol. Plant Microbe Interact.* 18 710–721. 10.1094/MPMI-18-0710 16042017

[B5] Bovie C. Ongena M. Thonart P. Dommes J. (2004). Cloning and expression analysis of cDNAs corresponding to genes activated in cucumber showing systemic acquired resistance after BTH treatment. *BMC Plant Biol.* 4:15. 10.1186/1471-2229-4-15 15331019 PMC516775

[B6] Caracuel Z. Martínez-Rocha A. L. Di Pietro A. Madrid M. P. Roncero M. I. G. (2005). Fusarium oxysporum gas1 encodes a putative β-1,3-glucanosyltransferase required for virulence on tomato plants. *Mol. Plant Microbe Interact.* 18 1140–1147. 10.1094/MPMI-18-1140 16353549

[B7] Constantin M. E. de Lamo F. J. Vlieger B. V. Rep M. Takken F. L. (2019). Endophyte-mediated resistance in tomato to *Fusarium oxysporum* is independent of ET, JA, and SA. *Front. Plant Sci.* 10:979. 10.3389/fpls.2019.00979 31417594 PMC6685397

[B8] de Boer W. Verheggen P. Klein Gunnewiek P. J. A. Kowalchuk G. A. van Veen J. A. (2003). Microbial community composition affects soil fungistasis. *Appl. Environ. Microbiol.* 69 835–844. 10.1128/AEM.69.2.835-844.2003 12571002 PMC143609

[B9] de Lamo F. J. Šimkovicová M. Fresno D. H. de Groot T. Tintor N. Rep M. (2020). Pattern-triggered immunity restricts host colonization by endophytic fusaria, but does not affect endophyte-mediated resistance. *Mol. Plant Pathol.* 18 1–12. 10.1111/mpp.13018 33205901 PMC7814963

[B10] Di Pietro A. D. García-Maceira F. I. Méglecz E. Roncero I. G. (2001). A MAP kinase of the vascular wilt fungus *Fusarium oxysporum* is essential for root penetration and pathogenesis. *Mol. Microbiol.* 39 1140–1152. 10.1111/j.1365-2958.2001.02307.x 11251832

[B11] Diallinas G. Kanellis A. K. (1994). A phenylalanine ammonia-lyase gene from melon fruit: cDNA cloning, sequence and expression in response to development and wounding. *Plant Mol. Biol.* 26 473–479. 10.1007/BF00039557 7948894

[B12] Duyvesteijn R. G. E. Wijk R. V. Boer Y. Rep M. Cornelissen B. J. C. Haring M. A. (2005). Frp1 is a Fusarium oxysporum F-box protein required for pathogenicity on tomato. *Mol. Microbiol.* 57 1051–1063. 10.1111/j.1365-2958.2005.04751.x 16091043

[B13] Edel-Hermann V. Lecomte C. (2019). Current status of Fusarium oxysporum formae speciales and races. *Phytopathology* 109 512–530. 10.1094/PHYTO-08-18-0320-RVW 30461350

[B14] Fravel D. Olivain C. Alabouvette C. (2003). Fusarium oxysporum and its biocontrol. *New Phytol.* 157 493–502. 10.1046/j.1469-8137.2003.00700.x 33873407

[B15] Fuchs J.-G. Moënne-Loccoz Y. Défago G. (1997). Nonpathogenic Fusarium oxysporum strain Fo47 induces resistance to *Fusarium* wilt in tomato. *Plant Dis.* 81 492–496. 10.1094/PDIS.1997.81.5.492 30861928

[B16] García-Gutiérrez L. Zeriouh H. Romero D. Cubero J. de Vicente A. Pérez-García A. (2013). The antagonistic strain *Bacillus subtilis* UMAF6639 also confers protection to melon plants against cucurbit powdery mildew by activation of jasmonate- and salicylic acid-dependent defence responses. *Microb Biotechnol.* 6 264–274. 10.1111/1751-7915.12028 23302493 PMC3815921

[B17] Husaini A. M. Sakina A. Cambay S. R. (2018). Host-pathogen interaction in fusarium oxysporum infections: where do we stand? *Mol. Plant Microbe Interact.* 31 889–898. 29547356 10.1094/MPMI-12-17-0302-CR

[B18] Imazaki I. Kurahashi M. Iida Y. Tsuge T. (2007). Fow2, a Zn(II)2Cys6-type transcription regulator, controls plant infection of the vascular wilt fungus *Fusarium oxysporum*. *Mol. Microbiol.* 63 737–753. 10.1111/j.1365-2958.2006.05554.x 17302801

[B19] Inoue I. Ohara T. Namiki F. Tsuge T. (2001). Isolation of pathogenicity mutants of *Fusarium oxysporum* f. sp. melonis by insertional mutagenesis. *J. Gen. Plant Pathol.* 67 191–199. 10.1105/tpc.002576 12172028 PMC151471

[B20] Jain S. Akiyama K. Kan T. Ohguchi T. Takata R. (2003). The G protein β subunit FGB1 regulates development and pathogenicity in *Fusarium oxysporum*. *Curr. Genet.* 43 79–86. 10.1007/s00294-003-0372-9 12695847

[B21] Jain S. Akiyama K. Mae K. Ohguchi T. Takata R. (2002). Targeted disruption of a G protein α subunit gene results in reduced pathogenicity in *Fusarium oxysporum*. *Curr. Genet.* 41 407–413. 10.1007/s00294-002-0322-y 12228810

[B22] Kuroda K. Suzuki H. Tomikawa A. Tanaka K. Ito T. Yamamoto T. (2004). Biological control of Fusarium wilt of strawberry with carbide of brewer’s grains by previously infested with nonpathogenic Fusarium oxysporum. *Jpn. J. Phytopathol.* 70:246.

[B23] Larkin R. P. Fravel D. R. (1998). Efficacy of various fungal and bacterial biocontrol organisms for control of *Fusarium* wilt of tomato. *Plant Dis.* 82 1022–1028. 10.1094/PDIS.1998.82.9.1022 30856829

[B24] Larkin R. P. Fravel D. R. (1999). Mechanisms of action and dose-response relationships governing biological control of *Fusarium* wilt of tomato by nonpathogenic *Fusarium* spp. *Phytopathology* 89 1152–1161. 10.1094/PHYTO.1999.89.12.1152 18944639

[B25] Larkin R. P. Hopkins D. L. Martin F. N. (1996). Suppression of Fusarium wilt of watermelon by nonpathogenic *Fusarium oxysporum* and other microorganisms recovered from a disease-suppressive soil. *Phytopathology* 86 812–819. 10.1094/Phyto-86-812

[B26] Lemanceau P. Bakker P. A. H. M. De Kogel W. J. Alabouvette C. Schippers B. (1993). Antagonistic effect on nonpathogenic Fusarium oxysporum strain Fo47 and pseudobactin 358 upon pathogenic Fusarium oxysporum f. sp. dianthi. *Appl. Environ. Microb.* 59 74–82. 10.1128/aem.59.1.74-82.1993 16348860 PMC202057

[B27] Livak K. J. Schmittgen T. D. (2001). Analysis of relative gene expression data using real-time quantitative PCR and the 2−ΔΔ CT method. *Methods* 25 402–408. 11846609 10.1006/meth.2001.1262

[B28] Lockwood J. L. (1977). Fungistasis in soils. *Biol. Rev.* 52 1–43. 10.1006/meth.2001.1262 11846609

[B29] Louvet J. Rouxel F. Alabouvette C. (1976). Recherches sur la résistance des sols aux maladies. I. Mise en évidence de la nature microbiologique de la résistance d’un sol au développement de la fusariose vasculaire du melon. *Ann. Phytopathology* 8 425–436.

[B30] Matta A. (1989). “Induced resistance to fusarium wilt diseases,” in *Vascular Wilt Diseases of Plants – Basic Studies and Control*, eds Tjamos E. C. Beckman C. H. (Berlin: Springer), 175–196. 10.1007/978-3-642-73166-2_13

[B31] Metraux J. P. Burkhart W. Moyer M. Dincher S. Middlesteadt W. Williams S. (1989). Isolation of a complementary DNA encoding a chitinase with structural homology to a bifunctional lysozyme/chitinase. *Proc. Natl. Acad. Sci. U.S.A.* 86 896–900. 10.1073/pnas.86.3.896 2915985 PMC286585

[B32] Michielse C. B. Rep M. (2009). Pathogen profile update: *Fusarium oxysporum*. *Mol Plant Pathol* 10 311–324. 10.1111/j.1364-3703.2009.00538.x 19400835 PMC6640313

[B33] Michielse C. B. van Wijk R. Reijnen L. Manders E. M. Boas S. Olivain C. (2009). The nuclear protein Sge1 of *Fusarium oxysporum* is required for parasitic growth. *PLoS Pathog.* 5:e1000637. 10.1371/journal.ppat.1000637 19851506 PMC2762075

[B34] Minerdi D. Bossi S. Gullino M. L. Garibaldi A. (2009). Volatile organic compounds: a potential direct long-distance mechanism for antagonistic action of *Fusarium oxysporum* strain MSA 35. *Environ. Microbiol.* 11 844–854. 10.1111/j.1462-2920.2008.01805.x 19396945

[B35] Minerdi D. Moretti M. Gilardi G. Barberio C. Gullino M. L. Garibaldi A. (2008). Bacterial ectosymbionts and virulence silencing in a *Fusarium oxysporum* strain. *Environ. Microbiol.* 10 1725–1741. 10.1111/j.1462-2920.2008.01594.x 18397306

[B36] Mizuno S. Hirasawa Y. Sonoda M. Nakagawa H. Sato T. (2006). Isolation and characterization of three DREB/ERF-type transcription factors from melon (*Cucumis melo*). *Plant Sci.* 170 1156–1163. 10.1016/j.plantsci.2006.02.005

[B37] Namiki F. Matsunaga M. Okuda M. Inoue I. Nishi K. Fujita Y. (2001). Mutation of an arginine biosynthesis gene causes reduced pathogenicity in *Fusarium oxysporum* f. sp. melonis. *Mol. Plant Microbe Interact.* 14 580–584. 10.1094/MPMI.2001.14.4.580 11310747

[B38] Namiki F. Shiomi T. Kayamura T. Tsuge T. (1994). Characterization of the formae speciales of *Fusarium oxysporum* causing wilts of cucurbits by DNA fingerprinting with nuclear repetitive DNA sequences. *Appl. Environ. Microbiol.* 60 2684–2691. 10.1128/aem.60.8.2684-2691.1994 8085813 PMC201709

[B39] Ogawa K. Komada H. (1984). Biological control of *Fusarium* wilt of sweet potato by non-pathogenic *Fusarium oxysporum*. *Ann. Phytopathol. Soc. Jpn.* 50 1–9.

[B40] Olivain C. Trouvelot S. Binet M. N. Cordier C. Pugin A. Alabouvette C. (2003). Colonization of flax roots and early physiological responses of flax cells inoculated with pathogenic and nonpathogenic strains of Fusarium oxysporum. *Appl. Environ. Microbiol.* 69 5453–5462. 10.1128/AEM.69.9.5453-5462.2003 12957934 PMC194917

[B41] Pareja-Jaime Y. Martín-Urdíroz M. González Roncero M. I. González-Reyes J. A. Ruiz Roldán M. D. C. (2010). Chitin synthase-deficient mutant of Fusarium oxysporum elicits tomato plant defense response and protects against wild-type infection. *Mol. Plant Pathol.* 11 479–493. 10.1111/j.1364-3703.2010.00624.x 20618706 PMC6640515

[B42] Postma J. Luttikholt A. J. G. (1996). Colonization of carnation stems by a nonpathogenic isolate of *Fusarium oxysporum* and its effect on *Fusarium oxysporum* f. sp. dianthi. *Can. J. Bot.* 74 1841–1851. 10.1139/b96-221

[B43] Postma J. Rattink H. (1992). Biological control of *Fusarium* wilt of carnation with a nonpathogenic isolate of *Fusarium oxysporum*. *Can. J. Bot.* 70 1199–1205. 10.1139/b92-150

[B44] Rouxel F. Alabouvette C. Louvet J. (1979). Recherches sur la résistance des sols aux maladies. IV. Mise en évidence du rôle des *Fusarium* autochtones dans la résistance d’un sol à la fusariose vasculaire du melon. *Ann. Phytopathol.* 11 199–207.

[B45] Sarkar D. Rovenich H. Jeena G. Nizam S. Tissier A. Balcke G. U. (2019). The inconspicuous gatekeeper: endophytic *Serendipita vermifera* acts as extended plant protection barrier in the rhizosphere. *New Phytol.* 224 886–901. 10.1111/nph.15904 31074884

[B46] Silvar C. Merino F. Díaz J. (2009). Resistance in pepper plants induced by *Fusarium oxysporum* f. sp. lycopersici involves different defence-related genes. *Plant Biol.* 11 68–74. 10.1111/j.1438-8677.2008.00100.x 19121115

[B47] Tamietti G. Alabouvette C. (1986). Résistance des sols aux maladies: XIII – rôle des *Fusarium oxysporum* non pathogènes dans les mécanismes de résistance d’un sol de Noirmoutier aux fusarioses vasculaires. *Agronomie* 6 541–548.

[B48] Toussoun T. A. (1975). “Fusarium-suppressive soils,” in *Biology And Control Of Soil-Borne Plant Pathogen*, ed. Bruehl G. W. (St Paul, MN: American Phytopathological Society), 145–151.

[B49] Uknes S. Mauch-Mani B. Moyer M. Potter S. Williams S. Dincher S. (1992). Acquired resistance in *Arabidopsis*. *Plant Cell* 4 645–656. 10.1105/tpc.4.6.645 1392589 PMC160161

[B50] Vanden Wymelenberg A. J. Cullen D. Spear R. N. Schoenike B. Andrews J. H. (1997). Expression of green fluorescent protein in *Aureobasidium pullulans* and quantification of the fungus on leaf surfaces. *BioTechniques* 23 686–690. 10.2144/97234st01 9343693

