# Erratum: Biocontrol activity of nonpathogenic strains of *Fusarium oxysporum*: colonization on the root surface to overcome nutritional competition

**DOI:** 10.3389/fmicb.2023.1275135

**Published:** 2023-08-30

**Authors:** Frontiers Production Office

**Affiliations:** Frontiers Media SA, Lausanne, Switzerland

**Keywords:** Fusarium wilt disease, biocontrol, nonpathogenic *Fusarium oxysporum*, *F. oxysporum* f. sp. *melonis*, *F. oxysporum* f. sp. *lycopersici*, pathogenicity mutant, nutrient competition, rhizosphere

Due to a production error, in the third paragraph of the **Discussion** section, the phrase “strain MSA35” appeared as “strain stocktickerMSA35.”

The corrected sentence appears below.

“For example, the sesquiterpene volatile α-humulenone emitted by non-pathogenic *F. oxysporum* strain MSA35 represses the expression of pathogenicity genes in *F. oxysporum* (Minerdi et al., 2009).”

The publisher apologizes for this mistake. The original article has been updated.

