# Supplementary material for: Biocontrol Activity of Nonpathogenic Strains of Fusarium oxysporum: Colonization on the Root Surface to Overcome Nutritional Competition
Source: Front Microbiol. 2022 Jan 27;13:826677. doi: 10.3389/fmicb.2022.826677 (PMC8828976; doi:10.3389/fmicb.2022.826677)
Supplement: Supplementary file 1 [file Data_Sheet_1.pdf]

Table S1 | Pathogenicity-related genes previously identified in *Fusarium oxysporum* pathogens

Gene	Accession [a]	Description	Reference
FGA1	FOXG_09359	G protein α subunit	Jain et al., 2002
FGB1	FOXG_11532	G protein β subunit	Jain et al., 2003
FMK1	FOXG_08140	MAP kinase	Di Pietro et al., 2001
FRP1	FOXG_00058	F-box protein	Duyvesteijn et al., 2005
GAS1	FOXG_17750	β-1,3 glucanosyltransferase	Caracuel et al., 2005

[a] Broad Institute (www.broadinstitute.org/annotation/genome/fusarium_group/).

Table S2 | Primers used in this satudy

Name	Sequence (5′→3′)[a]	Purpose
Primers for detection of *F. oxysporum* strains in soil		
GFP-F	cacatgaagcagcacgactt	Detection of GFP gene
GFP-R	agttcaccttgatgccgttc	
dsRed-F	agttccagtacggctccaa	Detection of DsRed gene
dsRed-R	atgtccagcttggagtccacgta	
melonEF1a-F	gaggaaacttaaagacgtacccaa	Detection of elongation factor gene of melon
melonEF1a-R	agtggtgaagtttgtcagaatgat	
Primers for construction of transformation vectors		
Ptef-RFP	cctcggaggaggccatgtttgacggtgatgtatgga	Linearization of pTEFEGFP vector
Tgla-RFP	ccacctgttcctgtagacaatcaatccatttcgcta	
RFP-F	atggcctcctccgaggacgt	Amplification of DsRed
RFP-R	ctacaggaacaggtggtggc	
PtrpC_f	ggatgtccatcttcgaaacg	Amplification of *hph* cassette for disruption vector and selection of mutants
TtrpC_r	acccagaatgcacaggta	
FMK1-F	*atg*tctcgatcgaaccccccctaacg	Amplification of entire *FMK1* (FOXG_08140)
FMK1-R	*tta*cctcataatctcctggtagatc	
FMK1-hphF	ttgcgattttggtcttcgtttcgaagatggacatcc	Linearization of pGEM-T Easy containing entire *FMK1* gene
FMK1-hphR	aagcagcggatcgcgctacctgtgcattctgggt	
FMK1-5′	aagaccaaaatcgcaaaccttgaga	Selection of Δ*FMK1* strains
FMK1-3′	gcgcgatccgctgcttcccaggagg	
FGA1-F	*atg*ggctgcggaatgagcac	Amplification of entire *FGA1* (FOXG_09359)
FGA1-R	*tta*gataagaccacagagacgcagg	
FGA1-hphF	cattgctcgcattgctcgtttcgaagatggacatcc	Linearization of pGEM-T Easy containing entire *FGA1* gene
FGA1-hphR	ggcatgtaatcaggagtacctgtgcattctgggt	
FGA1-5′	agcaatgcgagcaatgttgtcgaa	Selection of Δ*FGA1* strains
FGA1-3′	ctcctgattacatgcctaacgacca	
FGB1-F	*atg*aactcccaaggcaacagtgat	Amplification of entire *FGB1* (FOXG_11532)
FGB1-R	*tta*gtatgcccagattttaagctaa	
FGB1-hphF	ctggtgacatgacctgcgtttcgaagatggacatcc	Linearization of pGEM-T Easy containing entire *FGB1* gene
FGB1-hphR	aatatcccacttcatatacctgtgcattctgggt	
FGB1-5′	caggtcatgtcaccagaggatgtta	Selection of Δ*FGB1* strains
FGB1-3′	tatgaagtgggatattgagacaggc	
FRP1-F	*atg*tcgagtatattctcattcagtg	Amplification of entire *FRP1* (FOXG_00058)
FRP1-R	*tta*tccgcatcgtcatcagtgaca	
FRP1-hphF	caaactgaagctcaaccgtttcgaagatggacatcc	Linearization of pGEM-T Easy containing entire *FRP1* gene
FRP1-hphR	ggtgcaaatgcaagtctacctgtgcattctgggt	
FRP1-5′	gttgagcttcagtttggcgaagaaa	Selection of Δ*FRP1* strains
FRP1-3′	gacttgcatttgcaccccagaacga	
GAS1-F	*atg*aagttttccgctgccattgt	Amplification of entire *GAS1* (FOXG_17750)
GAS1-R	*tta*cagaaggaccataccggcac	
GAS1-hphF	ttctttgaggactactcgtttcgaagatggacatcc	Linearization of pGEM-T Easy containing entire *GAS1* gene
GAS1-hphR	aagaagacaggaacagtacctgtgcattctgggt	
GAS1-5′	agtagtcctcaaagaacttggcctg	Selection of Δ*GAS1* strains
GAS1-3′	ctgttcctgtcttcttcgctgagta	

[a] Primers have 20 or 18 bases (underlined) that are identical to the sequences at the ends of the *hph* cassette.

Table S3 | Plasmids used in this study

Plasmid	Construct[a]	Reference or source
pSH75	*trpC (p)::hph::trpC (t)*; for fungal transformation	Kimura and Tsuge (1993) [b]
pII99	*trpC (p)::hph::trpC (t)*; for fungal transformation	Namiki et al. (2001)
pTEFEGFP	*TEF (p)::EGFP::gla (t)*; for generating GFP-expressing strains	Vanden Wymelenberg *et al.* (1997)
pTEFdsRed	*TEF (p)::DsRed::gla (t)*; for generating DsRed-expressing strains	This study
pGEM-T Easy	Cloning vector	Promega
pKOFMK1	The *hph* cassette was inserted within a 1.2-kb fragment of *FMK1* cloned in pGEM-T Easy; for disruption of *FMK1*	This study
pKOFGA1	The *hph* cassette was inserted within a 1.3-kb fragment of *FGA1* cloned in pGEM-T Easy; for disruption of *FGA1*	This study
pKOFGB1	The *hph* cassette was inserted within a 1.4-kb fragment of *FGB1* cloned in pGEM-T Easy; for disruption of *FGB1*	This study
pKOFRP1	The *hph* cassette was inserted within a 1.8-kb fragment of *FRP1* cloned in pGEM-T Easy; for disruption of *FRP1*	This study
pKOGAS1	The *hph* cassette was inserted within a 1.7-kb fragment of *GAS1* cloned in pGEM-T Easy; for disruption of *GAS1*	This study

[a] *trpC (p)* and *trpC (t)*, *Aspergillus nidulans trpC* promoter and terminator, respectively; *TEF (p)*, *Aureobasidium pullulans TEF* promoter; *gal (t)*, *A. nidulans gla* terminator.

[b] Kimura, N., Tsuge, T. 1993. Gene cluster involved in melanin biosynthesis of the filamentous fungus *Alternaria alternata*. J Bacteriol. 175, 4427-4435.

Table S4 | List of melon genes used for qPCR analysis

Gene	Function	Accession [a]	Primer name	Primer sequence (5'-3')	Reference
PR-1a	acidic chitinase	EU556704	PR1a-F<br>PR1a-R	ccgatatgccctccgattgt<br>ataagggcccccagaatggc	Uknes *et al*., 1992<br>García-Gutiérrez *et al*., 2013
PR-5a	acidic thaumatin-like protein	MU54015	PR5a-F<br>PR5a-R	ctggaagaggaaagtgtgct<br>catcaacaaggctcacatca	Uknes *et al*., 1992
CGT	acetylglucosamin yltransferase	MU51130	CGT1-F<br>CGT1-R	tgcacttgatcagcttctca<br>gtatgtcatcacaccatctcaa	Bovie *et al*., 2004
PR-8	acidic chitinase	MU43561	PR8-F<br>PR8-R	gagttattcactctcctccgc<br>gccagactcgatatcgaaatc	Metraux *et al*., 1989
ERF1	ethylene response factor 1	AB125975	ERF1-F<br>ERF1-R	cggattcaaactcgaattcga<br>aacgcggcgattgtcgttct	Mizuno *et al*., 2006
PAL1	phenylalanine ammonia-lyase	X76130	PAL-F<br>PAL-R	ccaagctccttaaccacaatgtcaca<br>gaaaaaggaaccaaatcaccagaagc	Diallinas and Kanellis, 1994
ACT	actin	AB033599	actin-F<br>actin-R	catggaattgtcagtaactgggat<br>ttgagtggtgcttcagtaagaagaa	Diallinas and Kanellis, 1994
HPH	hygromycin resistance gene	-	hph_qPCR_F<br>hph_qPCR_R	ataggtcaggctctcgctga<br>gatgtaggagggcgtggat	Mesarich et al., 2014 [b]

[a] From NCBI GenBank or Cucurbit Genomics Database (www.icugi.org)

[b] Mesarich C.H., Griffiths, S.A., van der Burgt, A., Okmen, B., Beenen, H. G., Etalo, D. W., Joosten, M. H., de Wit, P. J. (2014) Transcriptome sequencing uncovers the *Avr5* avirulence gene of the tomato leaf mold pathogen *Cladosporium fulvum*. *Mol. Plant Microbe Interact*. 27, 846-857. doi: 10.1094/MPMI-02-14-0050-R
