# Supplementary figures and images for: Biocontrol Activity of Nonpathogenic Strains of Fusarium oxysporum: Colonization on the Root Surface to Overcome Nutritional Competition
Source: Front Microbiol. 2022 Jan 27;13:826677. doi: 10.3389/fmicb.2022.826677 (PMC8828976; doi:10.3389/fmicb.2022.826677)

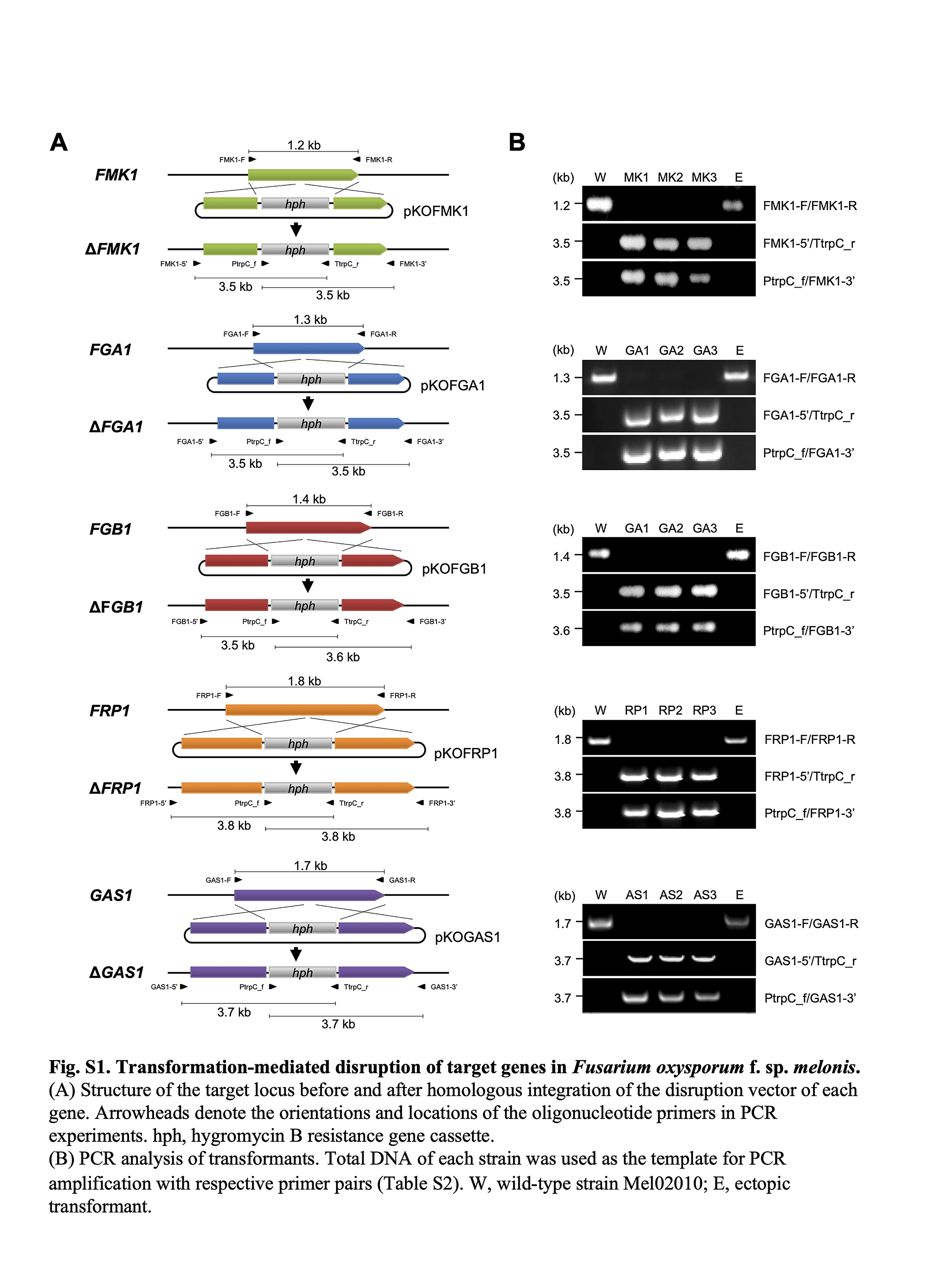

Supplement: Supplementary file 2 [file Image_1.TIFF]

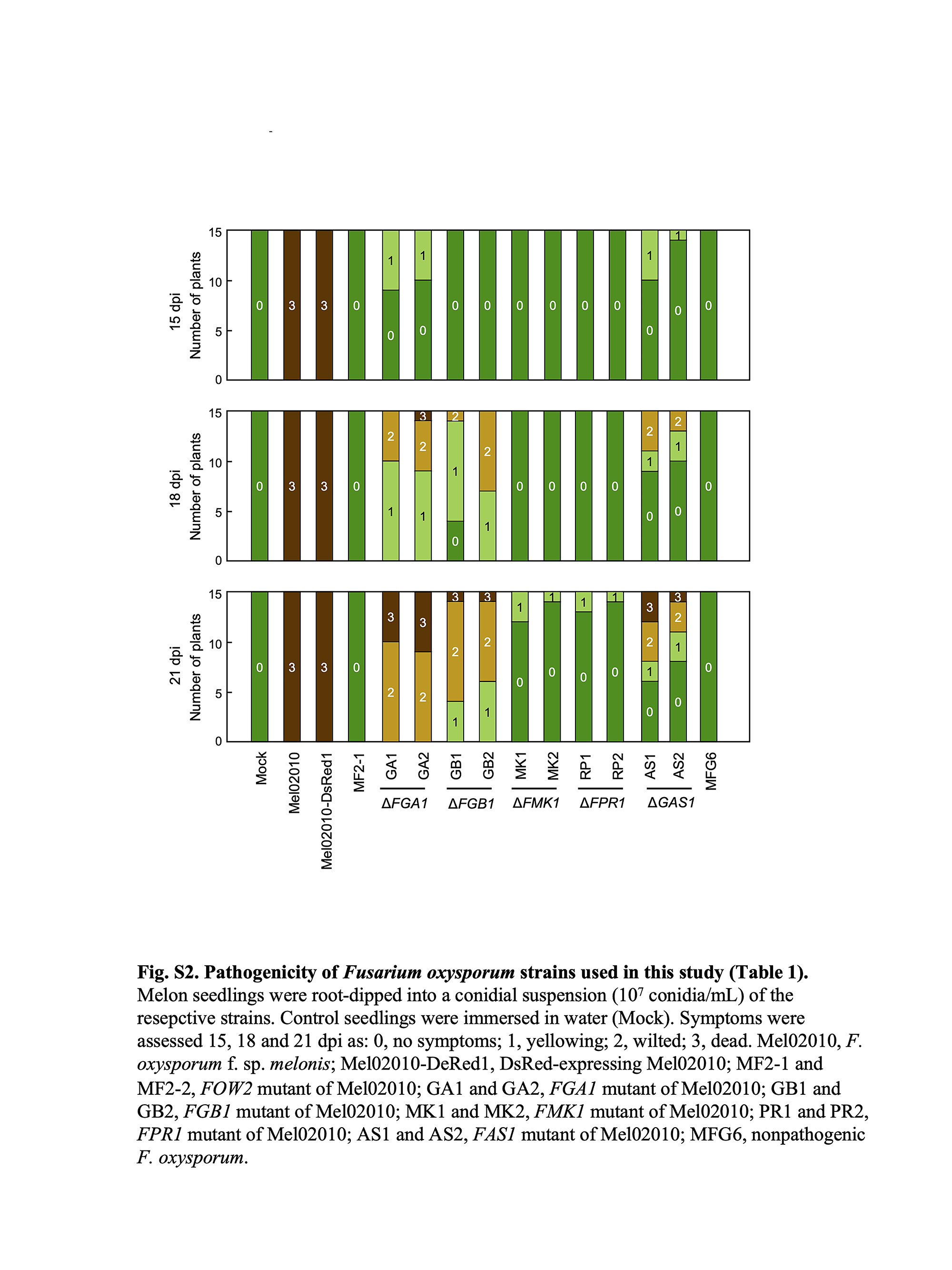

Supplement: Supplementary file 3 [file Image_2.TIFF]

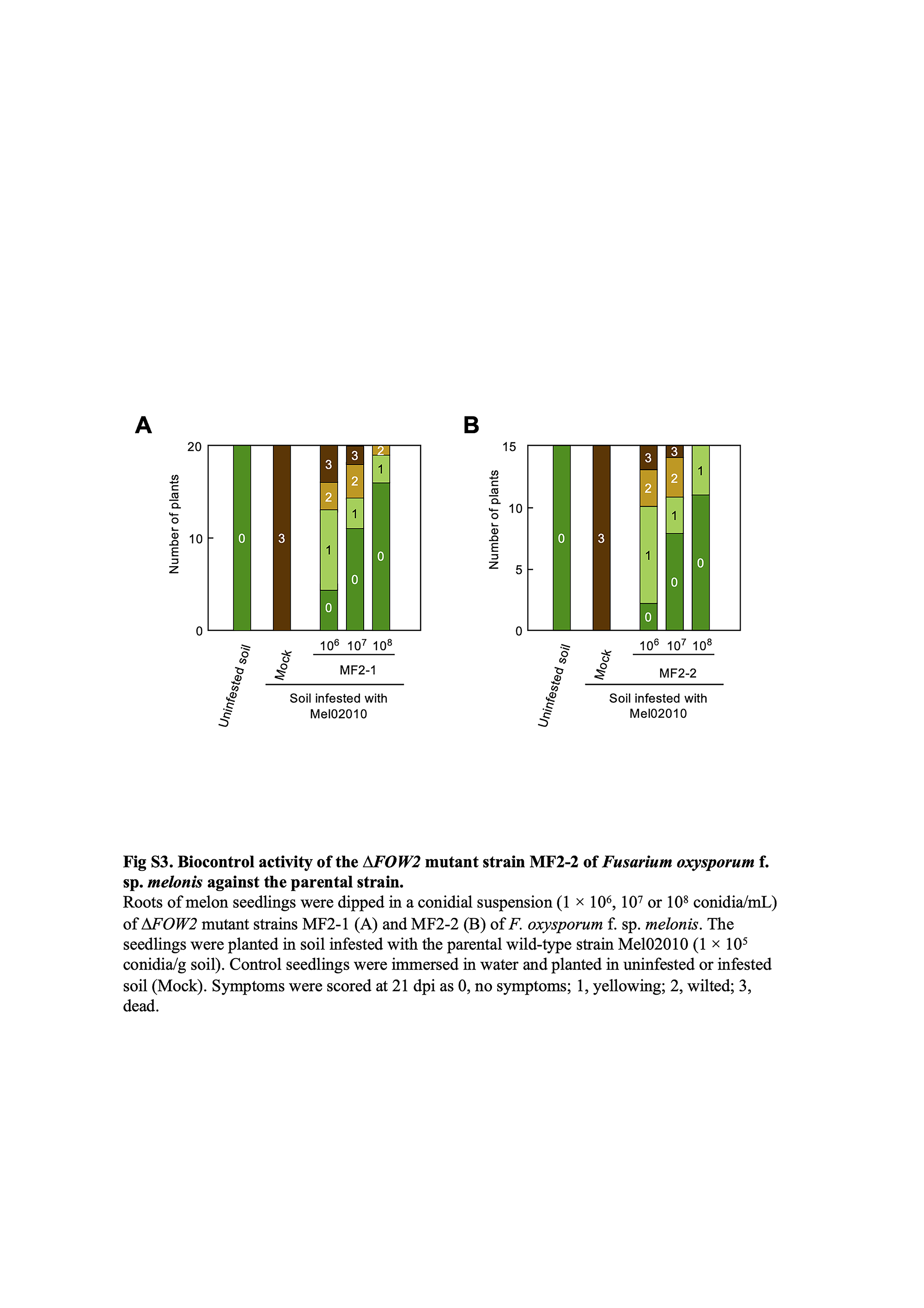

Supplement: Supplementary file 4 [file Image_3.TIFF]

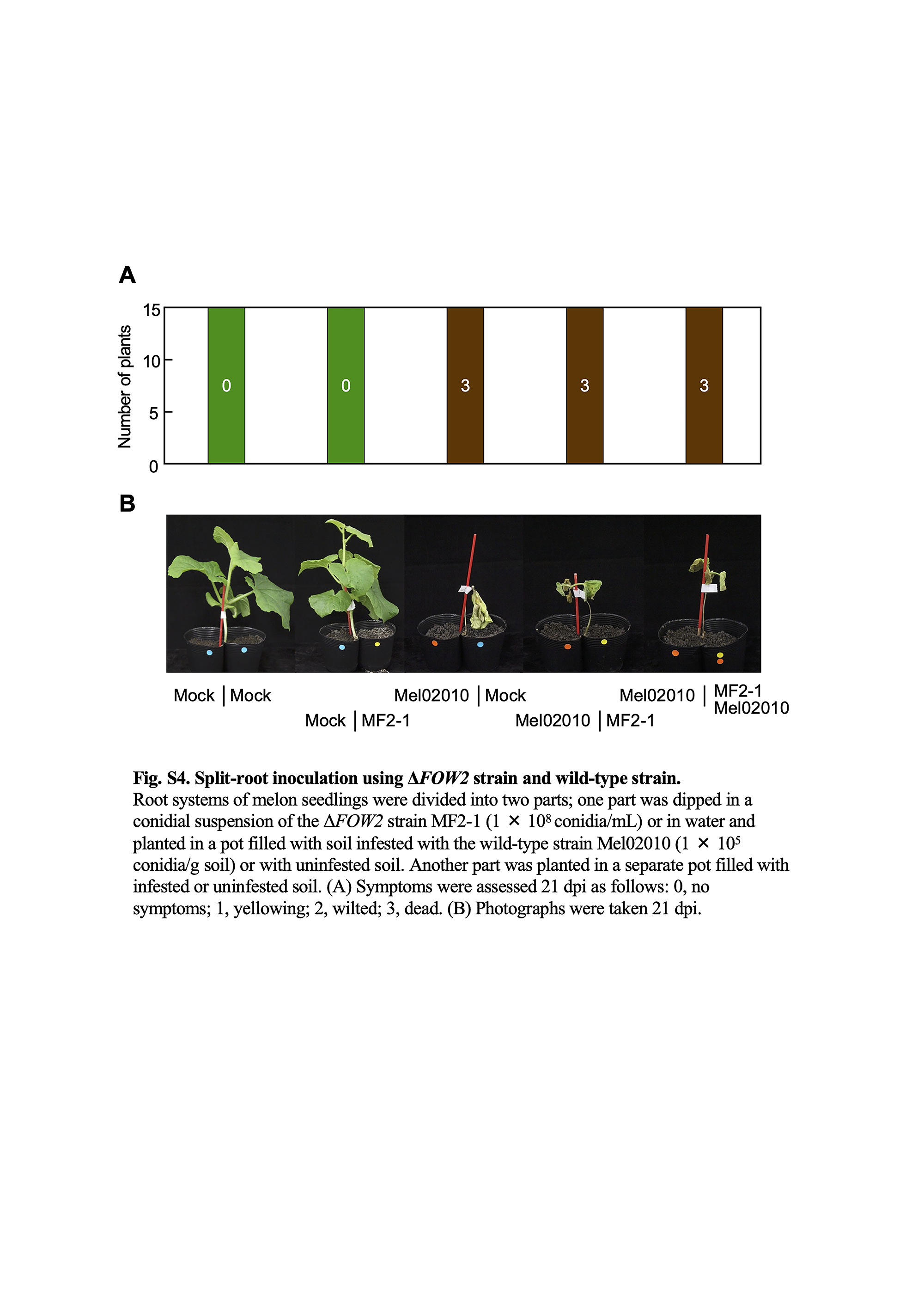

Supplement: Supplementary file 5 [file Image_4.TIFF]

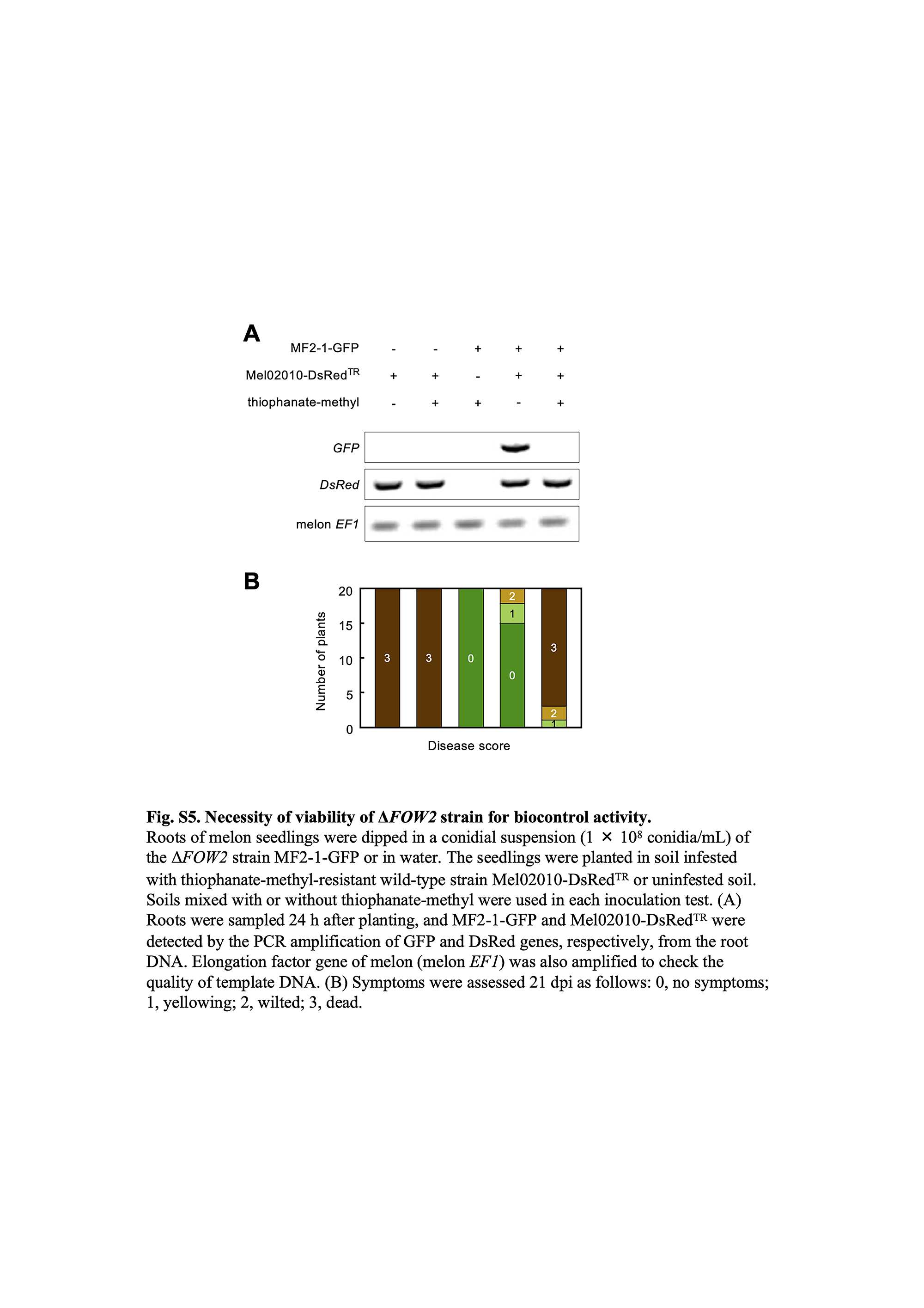

Supplement: Supplementary file 6 [file Image_5.TIFF]

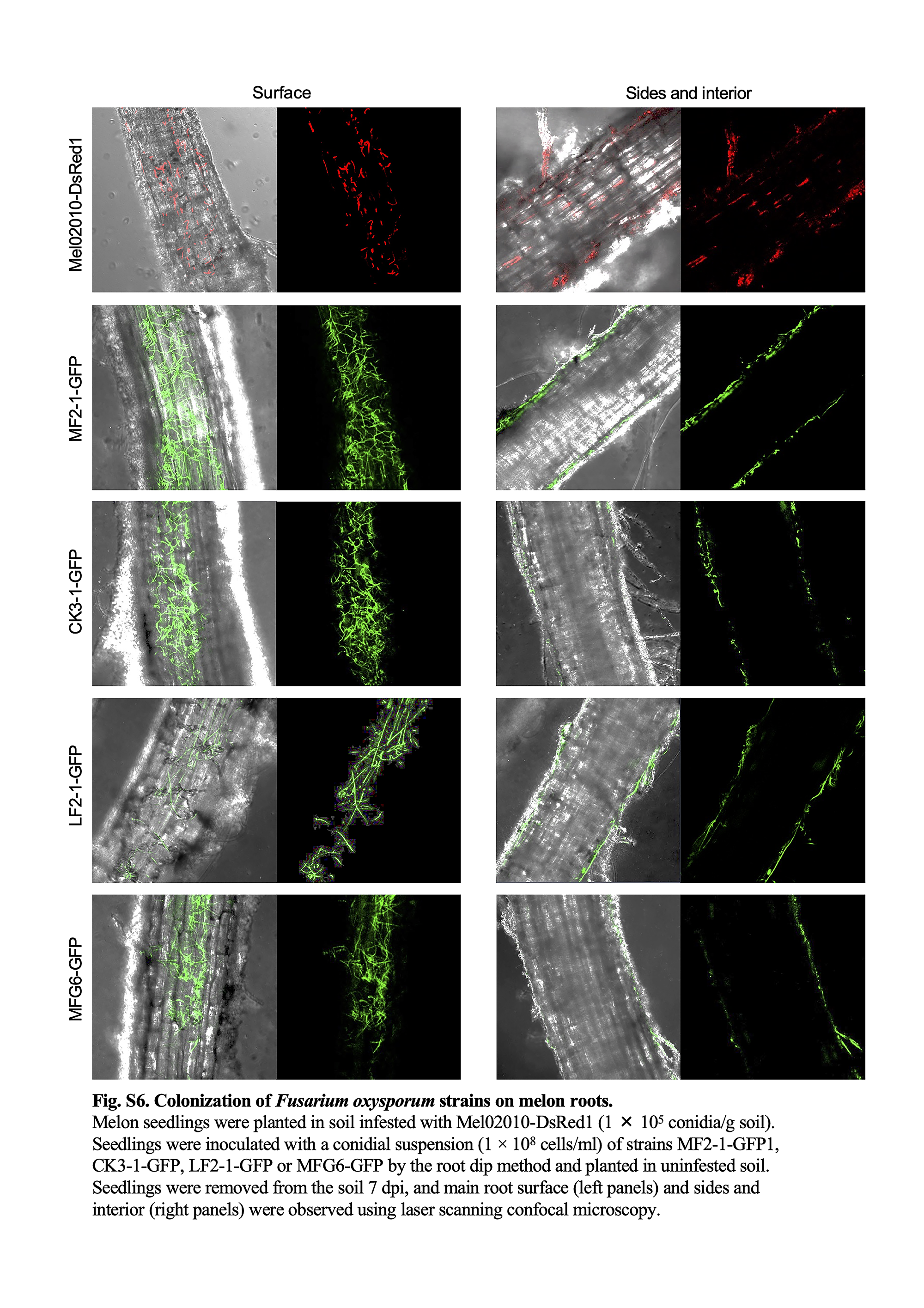

Supplement: Supplementary file 7 [file Image_6.TIFF]

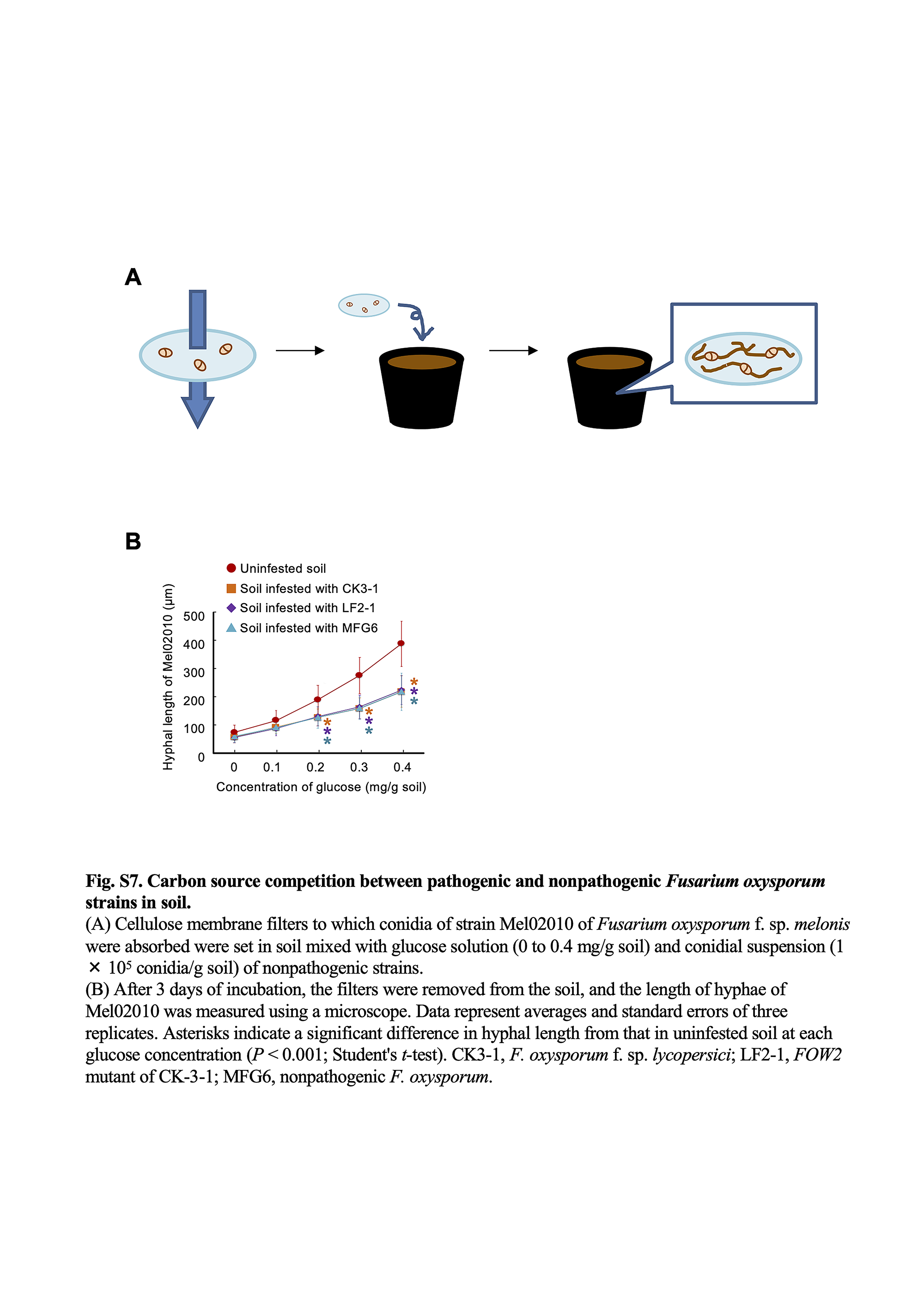

Supplement: Supplementary file 8 [file Image_7.TIFF]
